# Models of Bosch-Boonstra-Schaaf optic atrophy syndrome reveal genotype-phenotype correlations in brain structure and behavior

**DOI:** 10.1242/dmm.052426

**Published:** 2025-09-22

**Authors:** Johann G. Maass, Dominik Kamionek, Annabelle Mantilleri, Susanne Theiss, Laura Dötsch, Felix Franke, Tim Schubert, Jonas G. Scheck, Claudia Pitzer, Paolo Piovani, Michele Bertacchi, Olivier Deschaux, Anubhav Singh, Chun-An Chen, Henning Fröhlich, Michèle Studer, Christian P. Schaaf

**Affiliations:** ^1^ Institute of Human Genetics, Heidelberg University Clinic, 69120 Heidelberg, Germany; ^2^ Division of Genetics and Genomics, Boston Children's Hospital, Boston, MA 02115, USA; ^3^ Institute of Biology Valrose (iBV), University Côte d'Azur (UCA), CNRS 7277, Inserm 1091, Avenue Valrose 28, Nice 06108, France; ^4^ Neuroradiology Department, University Hospital Heidelberg, 69120 Heidelberg, Germany; ^5^ Clinical Cooperation Unit Translational Radiation Oncology, German Cancer Research Center (DKFZ), 69120 Heidelberg, Germany; ^6^ Interdisciplinary Neurobehavioral Core, Heidelberg University, 69120 Heidelberg, Germany; ^7^ Department of Molecular and Human Genetics, Baylor College of Medicine, Houston, TX 77030, USA

**Keywords:** NR2F1, ASD, Autism, Neurodevelopmental disorder, Bosch-Boonstra-Schaaf optic atrophy syndrome, BBSOAS, Social behavior

## Abstract

Bosch-Boonstra-Schaaf optic atrophy syndrome (BBSOAS) is a rare, autosomal dominant neurodevelopmental disorder caused by pathogenic variants in *NR2F1*, characterized by developmental delay, intellectual disability, optic nerve anomalies and autism spectrum disorder. Most pathogenic variants cluster within the highly conserved DNA-binding domain (DBD) or ligand-binding domain (LBD) of NR2F1 and are associated with variable clinical severity, suggesting a genotype-phenotype correlation. Although previous mouse models have provided important insights, comprehensive behavioral characterization remains limited. Here, we present two novel BBSOAS mouse models harboring patient-specific variants in the DBD (*Nr2f1^+/R139L^*) and LBD (Nr2f1^+/E397*^), alongside the established *Nr2f1^+/−^* model. We analyzed brain morphology and behavior to further expand the murine phenotype and investigate the genotype-phenotype correlation. We demonstrate that these models recapitulate key aspects of the BBSOAS phenotype, including deficits in cognition, social communication and motor function, and that the presence and severity of behavioral abnormalities are dependent on variant type. Our findings provide new evidence for a genotype-phenotype correlation associated with domain-specific *NR2F1* variants and establish a robust platform for future mechanistic and therapeutic studies.

## Teaser

**Summary:** Novel mouse models of Bosch-Boonstra-Schaaf optic atrophy syndrome with patient-specific *Nr2f1* variants show strong face validity and offer insights into underlying genotype-phenotype correlation.

## INTRODUCTION

Bosch-Boonstra-Schaaf optic atrophy syndrome [BBSOAS; Online Mendelian Inheritance in Man (OMIM) 615722; ORPHA 401777] is a rare, autosomal dominant neurodevelopmental disorder caused by heterozygous pathogenic variants in nuclear receptor subfamily 2 group F member 1 (*NR2F1*) (Bosch et al., 2014). Clinically, BBSOAS is characterized by developmental delay, intellectual disability, language impairments, optic nerve anomalies and autism spectrum disorder (Bertacchi et al., 2022). *NR2F1* encodes an orphan transcription factor that functions as a dimer and plays a critical role in neurogenesis. It is involved in cortical organization and regulates the balance between proliferation and differentiation (Tocco et al., 2021; Bertacchi et al., 2022; Cooney et al., 1992).

Most pathogenic variants in BBSOAS arise *de novo*. Although no recurrent hotspot variants have been identified, known pathogenic variants predominantly cluster within one of the two most highly conserved functional domains of the protein: the DNA-binding domain (DBD) and the ligand-binding domain (LBD) (Bertacchi et al., 2022). The syndrome is characterized by genetic heterogeneity and variable expressivity. For instance, cognitive impairment ranges from mild to severe intellectual disability, with some patients with BBSOAS being able to attend regular school with support, while others are nonverbal and wheelchair dependent (Bertacchi et al., 2022). A potential genotype-phenotype correlation was first proposed by Chen et al. (2016) and later investigated in a study of 92 patients with BBSOAS, which revealed that variants in the DBD are associated with a more severe clinical phenotype (Bertacchi et al., 2022). Developing and analyzing models that recapitulate this genotype-phenotype relationship is essential for advancing our understanding of BBSOAS.

Previous studies have evaluated individual mouse models carrying different *Nr2f1* variants, including a conditional knockout (cKO) in neocortical and hippocampal neurons, a constitutive heterozygous knockout (KO), and a heterozygous point mutation in the DBD (Tomassy et al., 2010; Contesse et al., 2019; Zhang et al., 2020; Chen et al., 2020; Armentano et al., 2007; Tocco et al., 2021). These models have provided insights into the impact of Nr2f1 deficiency on fundamental neurodevelopmental processes; however, several behavioral aspects of the BBSOAS phenotype in mice remain insufficiently characterized. Moreover, although only 16% of patients carry a deletion in *NR2F1*, most studies have relied on the *Nr2f1* KO model, which exhibits mild behavioral abnormalities (Bertacchi et al., 2022).

In this study, we generated two novel BBSOAS mouse models carrying patient-specific variants in the DBD (*Nr2f1^+/R139L^*) and the LBD (Nr2f1^+/E397*^). Both missense variants in the DBD and nonsense variants are associated with a dominant-negative effect (Bertacchi et al., 2022). Using these two new models, alongside the established *Nr2f1^+/−^* mouse model, we analyzed brain morphology throughout postnatal development and conducted a comprehensive battery of behavioral tests targeting key domains affected in individuals with BBSOAS, including cognition, social communication and motor function. Notably, we identified anomalies in the ultrasonic vocalizations (USVs) across all three mouse models, along with motor and cognitive impairments, which were most pronounced in mice carrying the DBD variant. Our results show that these models recapitulate the BBSOAS phenotype and further support the proposed genotype-phenotype correlation (Bertacchi et al., 2022).

These mouse models represent a significant addition to BBSOAS research, providing critical insights into genotype-phenotype relationships of the disorder and contributing to the foundation for future therapeutic developments. Moreover, they offer the scientific community novel murine models for studying autistic-like phenotypes, anxiety and other neurodevelopmental abnormalities.

## RESULTS

### BBSOAS mouse models exhibit reduced body weight at several stages of development

**Fig. 1.**
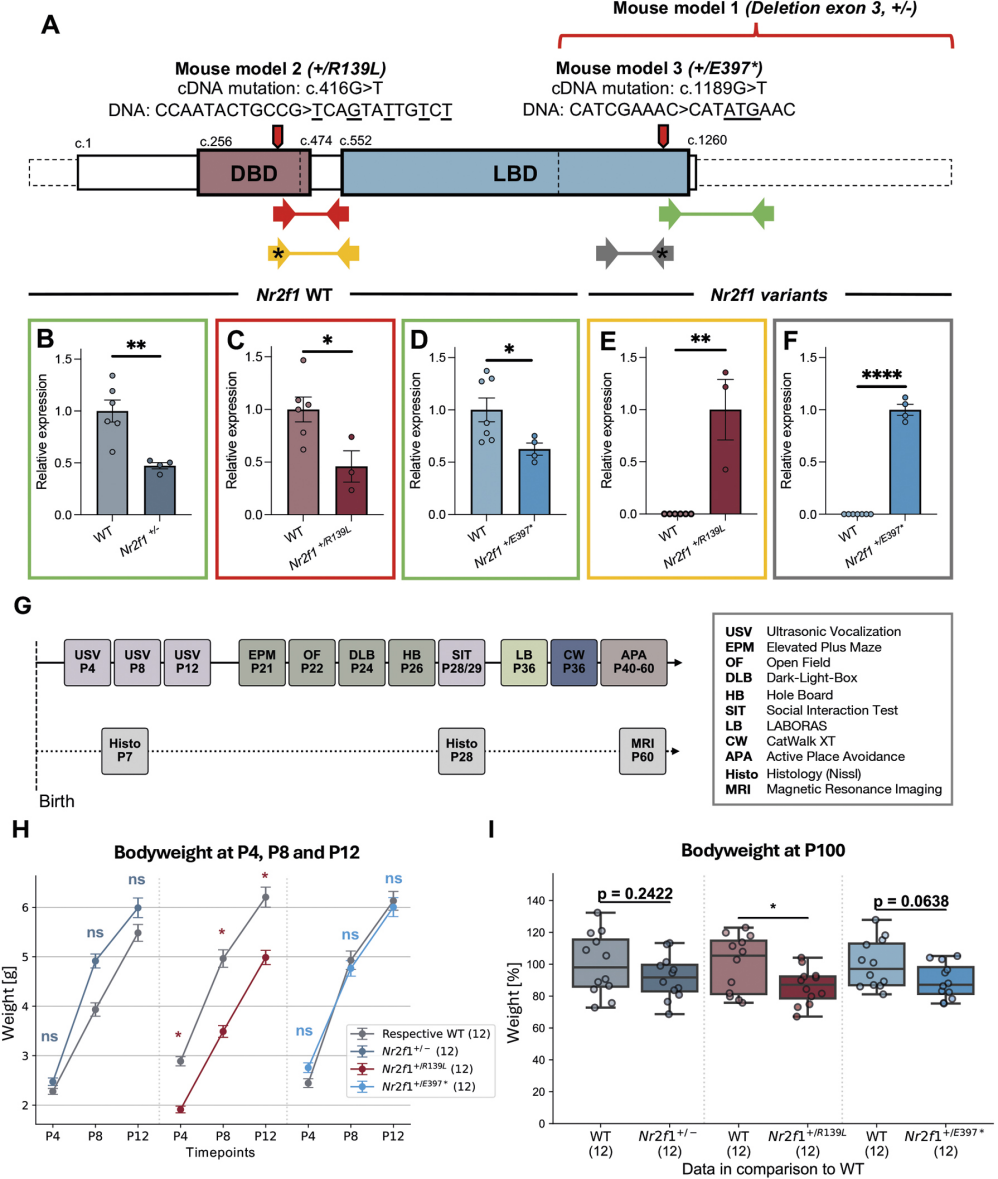
**Overview of the project, transcript quantification and body weight analysis.** (A) Schematic representation of *Nr2f1* mRNA with annotated domains. The DNA-binding domain (DBD) and the ligand-binding domain (LBD) are shown. Exons and exon-exon junctions are indicated by dashed line boxes and vertical dashed lines, respectively. The patient-specific genetic variants for the Bosch-Boonstra-Schaaf optic atrophy syndrome (BBSOAS) mouse models assessed in this paper are annotated. Primer pairs used for *Nr2f1* transcript detection are depicted and color coded with the boxes in B-F (Table S1). Primers marked with asterisks specifically recognize the mutant allele. (B-F) Real-time qRT-PCR analysis of *Nr2f1* expression (total expression and expression of specific variants) in whole-brain extracts from embryonic day (E)14.5 mouse embryos. Bars indicate s.e.m. *Nr2f1* wild-type (WT) allele expression is reduced in *Nr2f1^+/−^* (B), *Nr2f1^+/R139L^* (C) and Nr2f1^+/E397*^ (D) genotypes. The mutant allele is detected exclusively in *Nr2f1^+/R139L^* (E) and Nr2f1^+/E397*^ (F) embryos. Datapoints are normalized to the respective heterozygous mean. (G) The experimental timeline outlines the sequence of behavioral tests and brain morphology analysis conducted from postnatal day (P)4 to P60. Behavioral assessments were performed in two cohorts. Cohort sizes were progressively reduced by randomly excluding litters as the tests became more time intensive. Brain morphology analyses were carried out in three separate cohorts for each experiment. (H) *Nr2f1^+/R139L^* neonates exhibit reduced body weight at P4, P8 and P12. Bars indicate s.e.m. (I) *Nr2f1^+/R139L^* mice show reduced body weight at P100. Datapoints are normalized to the respective WT mean. Box plots represent the median and 25-75th percentiles; whiskers indicate the range of the data. ns, not significant; *P≤0.05, **P≤0.01, ****P≤0.0001; Student's *t*-test.

To investigate the effects of patient-specific *Nr2f1* variants on brain development and behavior, we compared three BBSOAS mouse models (Fig. 1A). The first model harbors a deletion of the third exon and the polyA tail, resulting in a functional null allele (*Nr2f1*^+/–^) (Armentano et al., 2006). The second model carries a missense mutation in the DBD (*Nr2f1^+/R139L^*) corresponding to a variant identified in a severely affected patient with BBSOAS (Chen et al., 2016). The third model features a nonsense variant in the LBD (Nr2f1^+/E397*^), which has also been reported in a patient (Jurkute et al., 2021). Transcript analysis of *Nr2f1* with mutation-specific primers revealed either reduced expression of the wild-type (WT) allele (Fig. 1B-D) or increased expression of the mutant allele (Fig. 1E,F). All three mouse models were subjected to an identical behavioral testing timeline, assessing various aspects of murine behavior (Fig. 1G). In addition, brain morphology was analyzed at different developmental stages (Fig. 1G).

The body weight of *Nr2f1^+/−^*, *Nr2f1^+/R139L^* and Nr2f1^+/E397*^ mice and their corresponding WT littermates was recorded at six developmental time points [postnatal day (P)4, P8, P12, P20, P30 and P100]. Although the weights of *Nr2f1*^+/−^ and Nr2f1^+/E397*^ animals did not differ from that of WT animals (Fig. 1H,I), *Nr2f1^+/R139L^* mice showed significantly lower body weight at all measured time points (except P30) (Fig. 1H,I; Fig. S1 and Table S6). At P100, both male and female Nr2f1^+/E397*^ mice and *Nr2f1^+/−^* males showed a sex-specific decrease in body weight (Fig. S1). Together, these findings suggest that different *Nr2f1* variants differentially affect growth, with the DBD missense variant exerting a more substantial and persistent impact on body weight.

### BBSOAS mouse models exhibit genotype-dependent alterations in brain morphology

**Fig. 2.**
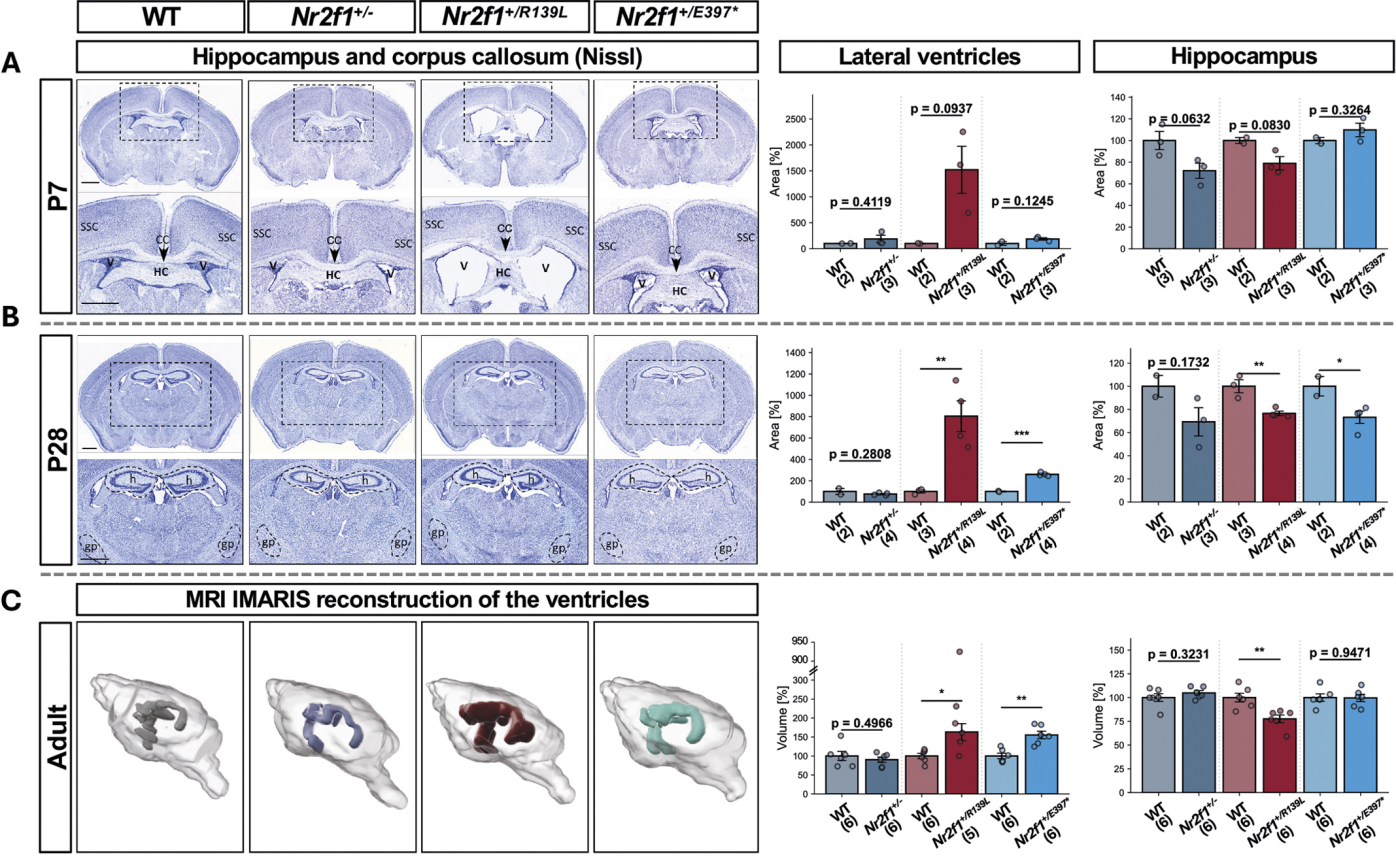
**Brain morphology in P7, P28 and adult mice.** (A) Nissl staining of *Nr2f1^+/−^*, *Nr2f1^+/R139L^* and Nr2f1^+/E397*^ brains. CC, corpus callosum; HC, hippocampal commissure; SCC, somatosensory cortex; V, ventricles. As shown in the quantifications on the right, *Nr2f1^+/R139L^* mice tend to have enlarged lateral ventricles. (B) Nissl staining of P28 *Nr2f1^+/−^*, *Nr2f1^+/R139L^* and Nr2f1^+/E397*^ brains. gp, globus pallidus; h, hippocampus. As shown in the quantifications on the right, *Nr2f1^+/R139L^* and Nr2f1^+/E397*^ mice have statistically enlarged lateral ventricles and a reduced hippocampal size. (C) 3D reconstructions (IMARIS) of representative brains and the ventricular system from MRI scans of 2-month-old WT, *Nr2f1^+/−^*, *Nr2f1^+/R139L^* and Nr2f1^+/E397*^ mice. As shown in the quantifications on the right, *Nr2f1^+/R139L^* and Nr2f1^+/E397*^ mice have statistically enlarged lateral ventricles. *Nr2f1^+/R139L^* mice show a reduced hippocampus. Bars indicate s.e.m. Datapoints are normalized to the respective WT mean. Box plots represent the median and 25-75th percentiles; whiskers indicate the range of the data. *P≤0.05, **P≤0.01, ***P≤0.001; Student's *t*-test. Scale bars: 1 mm.

To assess brain morphology, we performed histological analyses and magnetic resonance imaging (MRI) at multiple developmental stages (P7, P28 and adulthood) (Fig. 2). Although a total of 18 brain structures were examined, this section highlights the most prominent and consistent alterations observed across time points and *Nr2f1* mouse models. We observed that mice carrying pathogenic *Nr2f1* variants exhibited consistent changes in lateral ventricles and hippocampal size, replicated across multiple time points. At P7, *Nr2f1^+/R139L^* and Nr2f1^+/E397*^ mice showed a tendency for enlarged lateral ventricles; starting at P28, both lines exhibited a significant increase, with the effect being more pronounced in *Nr2f1^+/R139L^* mice (804% increase) (Fig. 2A,B). The same pattern could also be seen in adult *Nr2f1^+/R139L^* and Nr2f1^+/E397*^ mice, with an increase in the lateral ventricle size of 163% and 155%, respectively. Importantly, this change occurred without an overall change in total brain volume (Table S2A) and in the absence of a clear obstruction in the cerebroventricular system.

This enlargement of the lateral ventricles is accompanied by a reduction in surrounding structures, such as the hippocampus and the striatum (see Fig. 2 and Fig. S2). At P28, the hippocampal size was reduced by 23% in the *Nr2f1^+/R139L^* line and 26% in the Nr2f1^+/E397*^ line. This anomaly persisted into adulthood in *Nr2f1^+/R139L^* mice, with a 22% reduction. Additionally, all three lines (*Nr2f1^+/–^*, *Nr2f1^+/R139L^* and Nr2f1^+/E397*^) exhibited corpus callosum thinning at P28 (17%, 22% and 40% reductions, respectively) (Fig. S2). The findings regarding other brain structures (e.g. striatum and globus pallidus) are summarized in Table S2.

Overall, the three *Nr2f1* mouse lines display region-specific neuroanatomical abnormalities, including ventricular enlargement, hippocampal reduction and corpus callosum thinning, highlighting the critical role of Nr2f1 in embryonic and postnatal brain development.

### BBSOAS mouse models show autism-like behavior and genotype-dependent impairments in social interaction

Clinical studies indicate that patients with BBSOAS exhibit anomalies in social interactions, such as speech difficulties (80%) and autism spectrum disorder (39%) (Bertacchi et al., 2022). To investigate corresponding behavioral traits in mice, we analyzed neonatal ultrasonic vocalizations (USVs) and performed the social interaction test (SIT).

**Fig. 3.**
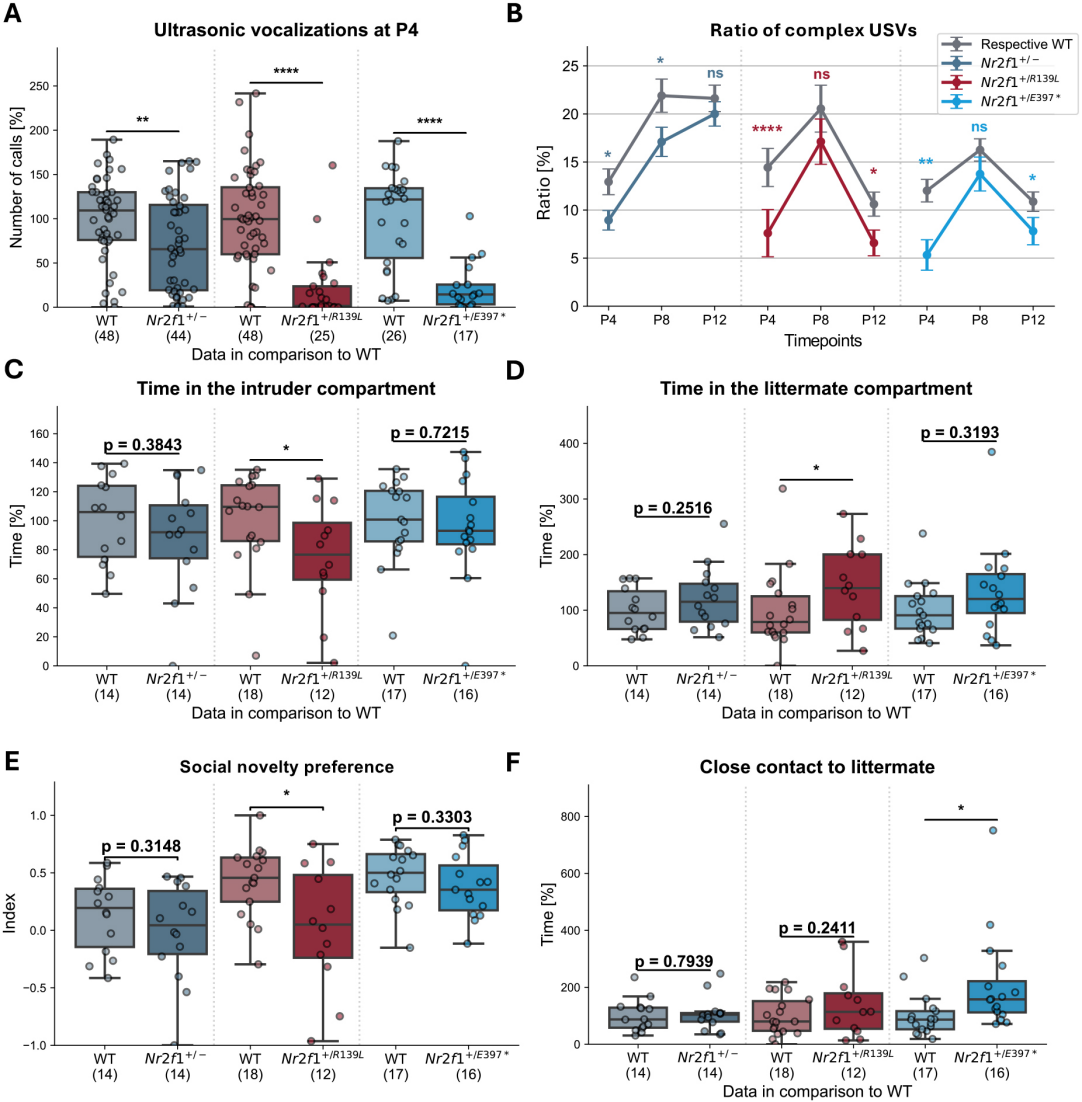
**Social and autism-like phenotype of mice with *Nr2f1* variants.** (A) Mice with *Nr2f1* variants emit fewer calls than WT littermates at P4. (B) Mice with *Nr2f1* variants show an altered call class profile, with a reduction in the prevalence of complex calls. Sample sizes for the *Nr2f1^+/−^* line were *n*=44, 41 and 40, and for the corresponding WT animals, *n*=48, 46 and 39, at P4, P8, and P12, respectively. For the *Nr2f1^+/R139L^* line, sample sizes were *n*=25, 23 and 23, with corresponding WT animals *n*=48, 45 and 42, at P4, P8 and P12, respectively. For the Nr2f1^+/E397*^ line, sample sizes were *n*=17, 17 and 16, and corresponding WT animals *n*=26, 26 and 26, at P4, P8 and P12, respectively. Error bars represent the s.e.m. (C,D) *Nr2f1^+/R139L^* mice spend less time in the compartment of the intruder (C) and more time in the compartment of their littermate (D) during the third trial of the social interaction test (SIT). (E) *Nr2f1^+/R139L^* mice have a lower social novelty preference index (‘TimeWithIntruder’ subtracted from ‘TimeWithLittermate’ divided by ‘TimeWithIntruder’ and ‘TimeWithLittermate’). (F) Nr2f1^+/E397*^ mice spend more time in the close contact zone with their littermate during the third trial of the SIT. ns, not significant; *P≤0.05, **P≤0.01, ****P≤0.0001; Student's *t*-test. Datapoints in A, C, D and F of each line are normalized to the respective WT mean. Box plots represent the median and 25-75th percentiles; whiskers indicate the range of the data.

Analyzing the USVs emitted by the pups provides insights into early social communication deficits and autism-like behavior (Scattoni et al., 2008; Takumi et al., 2020; Ey et al., 2013). All three genetic mouse models showed a reduction in the number of USVs at P4 (Fig. 3A). Compared to their WT littermates, the number of calls was reduced by 30%, 80% and 76% in *Nr2f1^+/−^*, *Nr2f1^+/R139L^* and Nr2f1^+/E397*^ mice, respectively. However, by P8 and P12, no significant differences in call frequency were observed (Table S6).

In addition to quantitative comparisons, we analyzed 11 call characteristics identified as biologically relevant (see Table S3) (Coffey et al., 2019). To illustrate these findings, a subset of the results is highlighted below, focusing on selected call features altered in more than one mouse line. At P4, all three mouse lines exhibited reduced average call duration, with *Nr2f1^+/−^* mice also showing reduced duration at P8. Call power and tonality were significantly diminished at P4 in both *Nr2f1^+/R139L^* and Nr2f1^+/E397*^ models. By P8, call characteristics in these two models were comparable to those in controls; however, at P12, both *Nr2f1^+/R139L^* and Nr2f1^+/E397*^ mice displayed a reduced slope of call frequency.

Call characteristics, such as duration, frequency changes and complexity, can be integrated into a unified classification system. In this study, we applied a modified version of the categories originally described by Scattoni et al. (2008). Using the deep learning algorithm and AI developed by Herdt et al. (2024), we classified calls into five distinct categories. Analysis of call types using this AI model, specifically built and trained for this task, revealed a significantly altered distribution of vocalization classes at P4, P8 and P12, with a pronounced shift toward simpler categories (classes 1, 2 and 5), as illustrated in Fig. 3B. The proportion of complex calls (those featuring a frequency step or two simultaneous frequencies) was reduced at P4 across all mouse models. At P8, only *Nr2f1^+/−^* mice displayed a significant reduction, whereas at P12, both *Nr2f1^+/R139L^* and Nr2f1^+/E397*^ mice exhibited fewer complex calls, as assessed by the deep learning model developed by Herdt et al. (2024) (Fig. 3B).

In addition, we used the SIT approach to evaluate the social interest of early adolescent mice. In the third trial of the SIT, mice can choose to spend time in a compartment with a novel intruder animal or a littermate (for illustration of the setup, refer to Fig. S3). Mice typically show strong social interest in novel conspecifics; however, *Nr2f1^+/R139L^* mice spent 30% less time in the intruder compartment during trial 3 of the 5-min test (Fig. 3C) and 41% more time in the littermate compartment (Fig. 3D), thus displaying reduced social interest in novel animals (Harro, 2023). These mice also spent less time in the close-contact zone with the intruder (Fig. S3). Consistently, *Nr2f1^+/R139L^* mice showed a reduced social novelty preference index (Fig. 3E) (Baronio et al., 2015). All four effects were observed exclusively in the *Nr2f1^+/R139L^* mouse line, suggesting a deficit in novelty preference and reduced social motivation. Notably, an increase in time spent in the close-contact zone with the littermate was observed only for the Nr2f1^+/E397*^ mouse line compared to their WT littermates (Fig. 3F). This could imply heightened attachment to familiar conspecifics, potentially due to social anxiety or a reduced motivation to engage with novel individuals. Finally, *Nr2f1*^+/−^ mice did not display significant alterations during the SIT test.

Our data show that Nr2f1-deficient mouse models exhibit altered social behavior at different developmental stages, and that the nature of these alterations depends on the type of *Nr2f1* mutation.

### BBSOAS mouse models show genotype-dependent alterations in anxiety and exploratory behavior

**Fig. 4.**
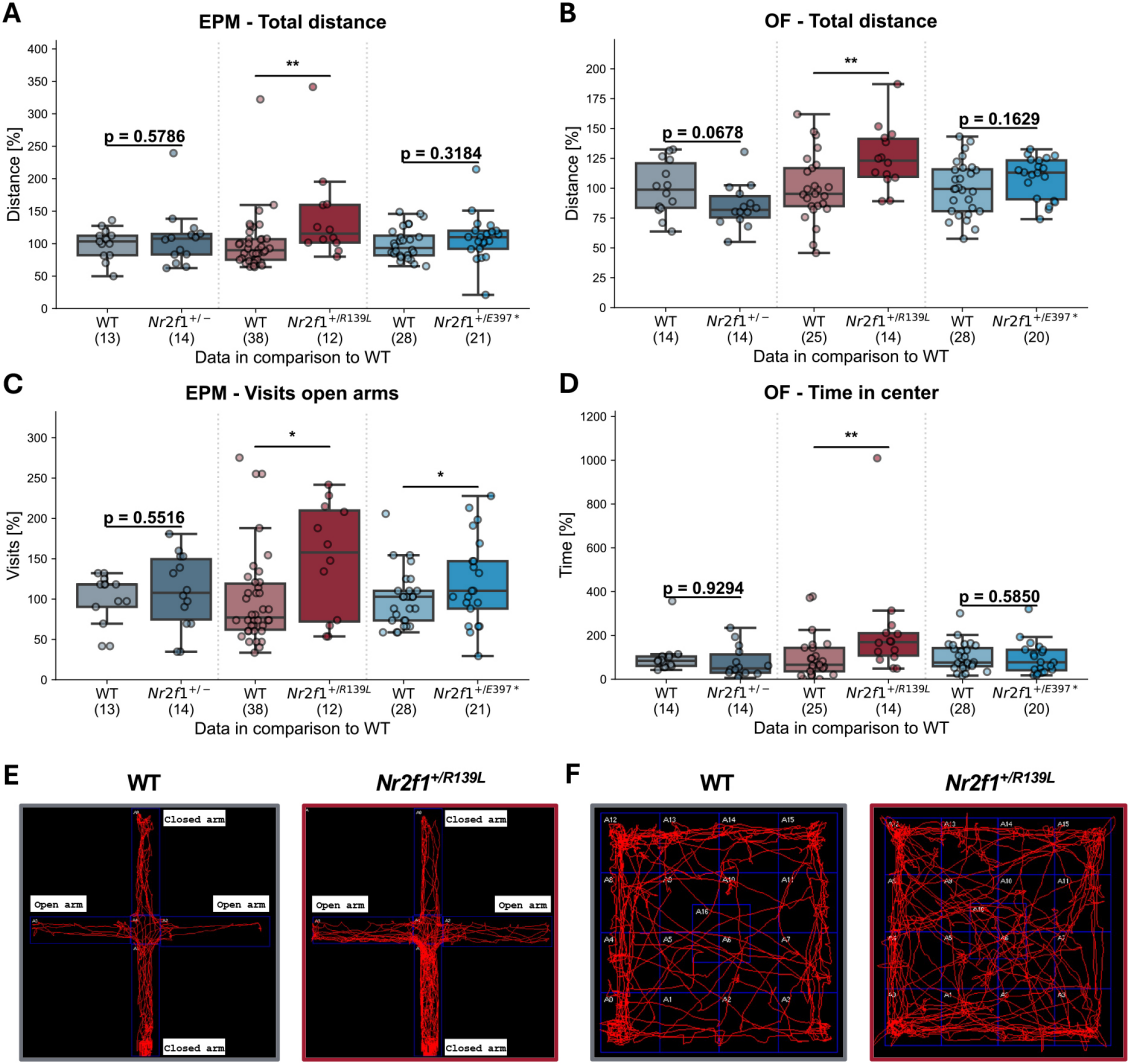
**Anxiety and exploratory behavior evaluated by the open field (OF) and elevated plus maze (EPM) tests in mouse models for BBSOAS.** (A,B) *Nr2f1^+/R139L^* show increased movement over the 10-min test period compared to their respective WT littermates in EPM (A) and OF (B) tests. (C) *Nr2f1^+/R139L^* and Nr2f1^+/E397*^ mice visit the open arms of the EPM arena more often than WT littermates. (D) *Nr2f1^+/R139L^* mice spend more time in the center of the OF arena and show increased movement over the 10-min test period compared to WT littermates. *P≤0.05, **P≤0.01; Student's *t*-test. Datapoints for each line are shown, normalized to the respective WT mean. Box plots represent the median and 25-75th percentiles; whiskers indicate the range of the data. (E,F) Representative track maps for WT and *Nr2f1^+/R139L^* mice in the EPM (E) and OF (F). The selected animals have individual performance parameters close to the mean of their respective group.

Complementing our assessments of social communication, we further evaluated anxiety-related and exploratory behavior in BBSOAS mouse models using the elevated plus maze (EPM), open field (OF) and dark-light box (DLB) tests. The hole board (HB) test was used to assess repetitive behaviors. Neither the DLB nor the HB assay revealed significant differences between mutant animals and their WT littermates for any of the parameters analyzed (Table S6). In the EPM, *Nr2f1^+/R139L^* mice traveled a greater distance (30%) during the 10-min test period and spent more time in the open arms (40%), as shown in Fig. 4A and Fig. S3. In addition, both *Nr2f1^+/R139L^* and Nr2f1^+/E397*^ mice entered the open arms more frequently, by 40% and 27%, respectively (Fig. 4C), suggesting enhanced exploratory behavior or reduced anxiety-like traits in these models.

*Nr2f1^+/R139L^* mice exhibited a similar trend in the OF test, in which they traveled a greater distance (24%) and spent more time (218%) in the center of the arena (Fig. 4B,D) during the 10-min test period. Representative track maps of WT and *Nr2f1^+/R139L^* animals are shown in Fig. 4E,F. In contrast, *Nr2f1^+/−^* mice did not show significant changes in anxiety or exploratory behavior.

In the 24-h home-cage monitoring test [Laboratory Animal Behavior Observation Registration and Analysis System (LABORAS)], which analyzes a wide range of behaviors, including anxiety and exploration, mutant and WT mice exhibited largely similar behavior, with only minor differences (Table S6). Specifically, *Nr2f1^+/E397^* mice showed a reduction in climbing distance and an increase in overall distance traveled. In contrast, *Nr2f1^+/R139L^* mice displayed an increased climbing frequency, while all other parameters remained unchanged.

Taken together, the increased time spent in the open arms of the EPM and the center of the OF, along with the increased total distance traveled and the higher number of entries into the open arms, can be interpreted as indicators of hyperactivity or reduced anxiety, respectively.

### BBSOAS mouse models show genotype-dependent motor function and coordination deficits

**Fig. 5.**
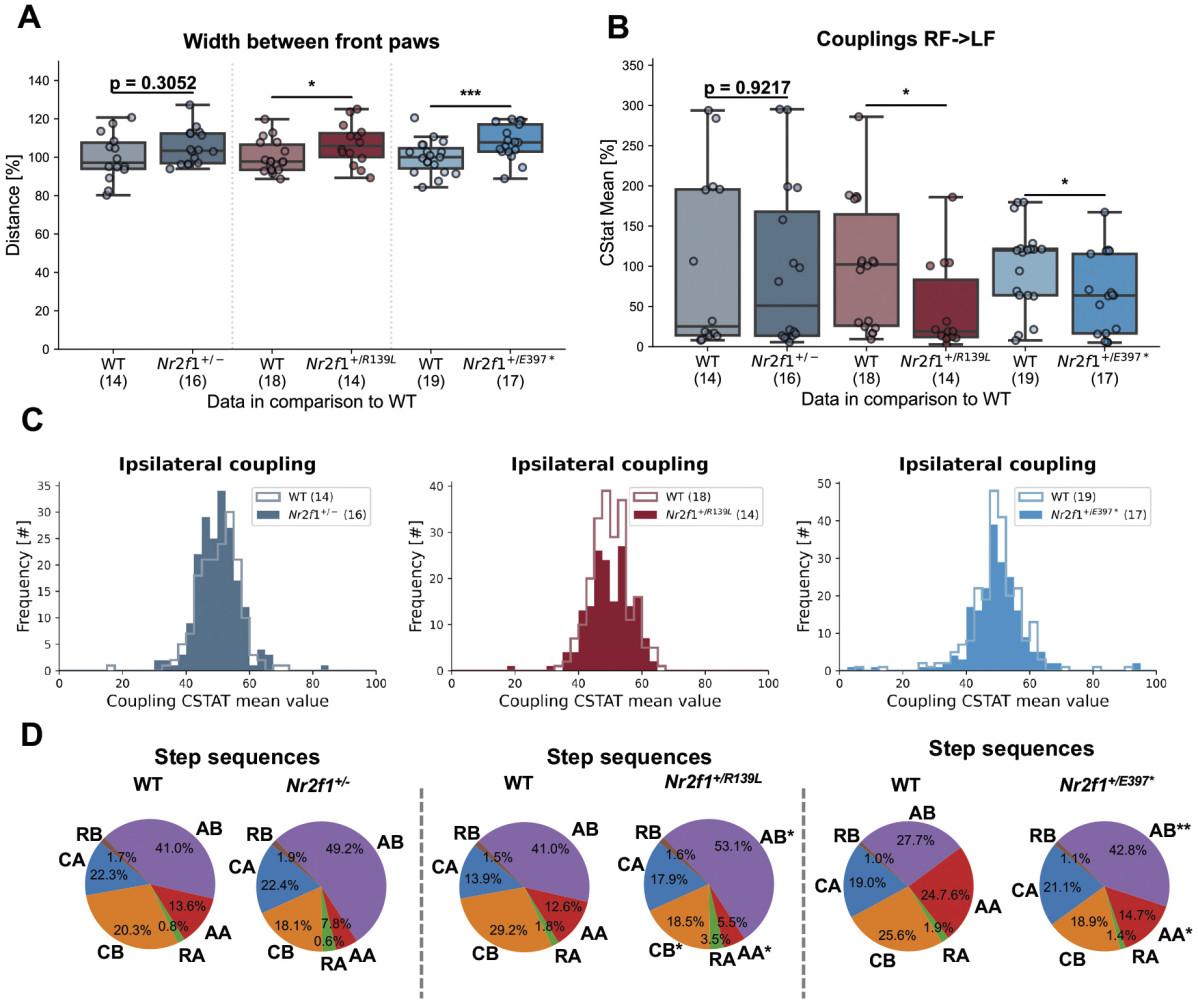
**Gait analysis using the CatWalk XT system.** (A) *Nr2f1^+/−^*, *Nr2f1^+/R139L^* and Nr2f1^+/E397*^ mice show an increase in the width between the front paws. (B) All mouse models display altered girdle coupling of the right-front (RF) paw to the left-hind (LF) paw. Datapoints for each line are shown, normalized to the respective WT mean. Box plots represent the median and 25-75th percentiles; whiskers indicate the range of the data. (C) Descriptive histograms showing the distribution of different coupling values for all four ipsilateral paw pairs for each mouse model. (D) Illustration of the step sequence type distribution for each line and genotype (WT and variant-carrying mice). Because of the data structure, no outlier analysis was done for the step sequence analysis. *P≤0.05, **P≤0.01, ***P≤0.001; Student's *t*-test.

Given previous findings in *Nr2f1* cKO mouse models and the motor impairments observed in patients with BBSOAS (Bertacchi et al., 2022; Tomassy et al., 2010), we further examined motor function, limb coordination and gait stability across all genotypes using the CatWalk XT (CW) system. We observed an increase in front paw width in *Nr2f1* mutants: +0.075 cm (not significant) in *Nr2f1^+/–^*, +0.094 cm (*P*≤0.05) in *Nr2f1^+/R139L^* and +0.099 cm (*P*≤0.001) in Nr2f1^+/E397*^ mice (Fig. 5A). However, the width between the hind paws remained unchanged (Fig. S4).

Beyond a wider front paw stance, stability can also be enhanced by reducing lateral paw support, which reflects the proportion of time the ipsilateral forelimb and hindlimb remain in contact with the walkway (e.g. left-front and left-hind paw). This parameter was significantly reduced in *Nr2f1^+/−^* and Nr2f1^+/E397*^ mice (Fig. S4). In contrast, diagonal (e.g. left front and right hind or right front and left hind) and girdle (e.g. left front and right front or left hind and right hind) support were not altered (Fig. S4).

To further assess locomotor stability, we analyzed temporal and spatial coordination and interlimb synchronization, using coupling parameters, which measure the timing difference between initial paw contacts (e.g. right-front paw and right-hind paw) as a percentage of the step cycle duration of the reference paw (e.g. right-front paw). Coupling was assessed for three specific paw pairings – diagonal, ipsilateral and girdle – across all 12 possible combinations. In mice, paw movements follow a consistent and precisely regulated timing pattern. For example, ipsilateral paw pairs (e.g. left front and left hind or right front and right hind) and girdle paw pairs typically exhibit coupling values around 50 CStat (circular mean of phase dispersion, i.e., the percentage timing offset between initial contacts of two paws within a step cycle), while diagonal paw pairs approach 0 or 100 CStat, respectively (Kloos et al., 2005). As shown in Fig. 5C and Fig. S5, overall coupling parameters across combined paw pair types did not significantly differ between mutant lines and WT controls. However, deviations in individual paw pair couplings indicated specific coordination deficits. Across the CW dataset, we observed a cumulative effect of these alterations, with no, two and eight parameters in the *Nr2f1^+/−^*, *Nr2f1^+/R139L^* and Nr2f1^+/E397*^ lines, respectively (Table S6). An example of impaired coupling in *Nr2f1^+/R139L^* and Nr2f1^+/E397*^ mice for the right-front and left-front paw is shown in Fig. 5B.

Quadrupeds adapt various step sequences (AA, AB, CA, CB and more; see Maricelli et al., 2016) depending on context and motor ability (Mendes et al., 2015). Although the total number of step sequences used remained unchanged (Fig. S4), *Nr2f1^+/R139L^* mice showed a 12.1% increase in AB usage and a 7.1% decrease in AA usage compared to WT littermates (Fig. 5D). Nr2f1^+/E397*^ mice exhibited a similar trend, with AB increasing by 15.1% and AA decreasing by 9.9% (Fig. 5D). No significant step sequence changes were observed in *Nr2f1^+/−^* mice.

Analysis of the regularity and precision of step sequences, as quantified by the regularity index, which integrates multiple parameters to assess interlimb coordination consistency, revealed no significant differences between groups (Fig. S4). Similarly, the parameter linear discriminant analysis (P_LDA_) score, which applies mathematical models to combine relevant gait parameters (Timotius et al., 2021), showed no significant variation in overall motor performance (Fig. S4).

Consistent with our observations during animal handling, the data indicate that all three mouse models exhibit mild, but evident, motor dysfunction. CW analysis showed that although overall performance, reflected in P_LDA_ scores or walking speed, is not severely compromised, specific parameters corresponding to coordination and balance are impaired in a genotype-dependent manner.

### Mice carrying *Nr2f1* variants exhibit impaired spatial learning

**Fig. 6.**
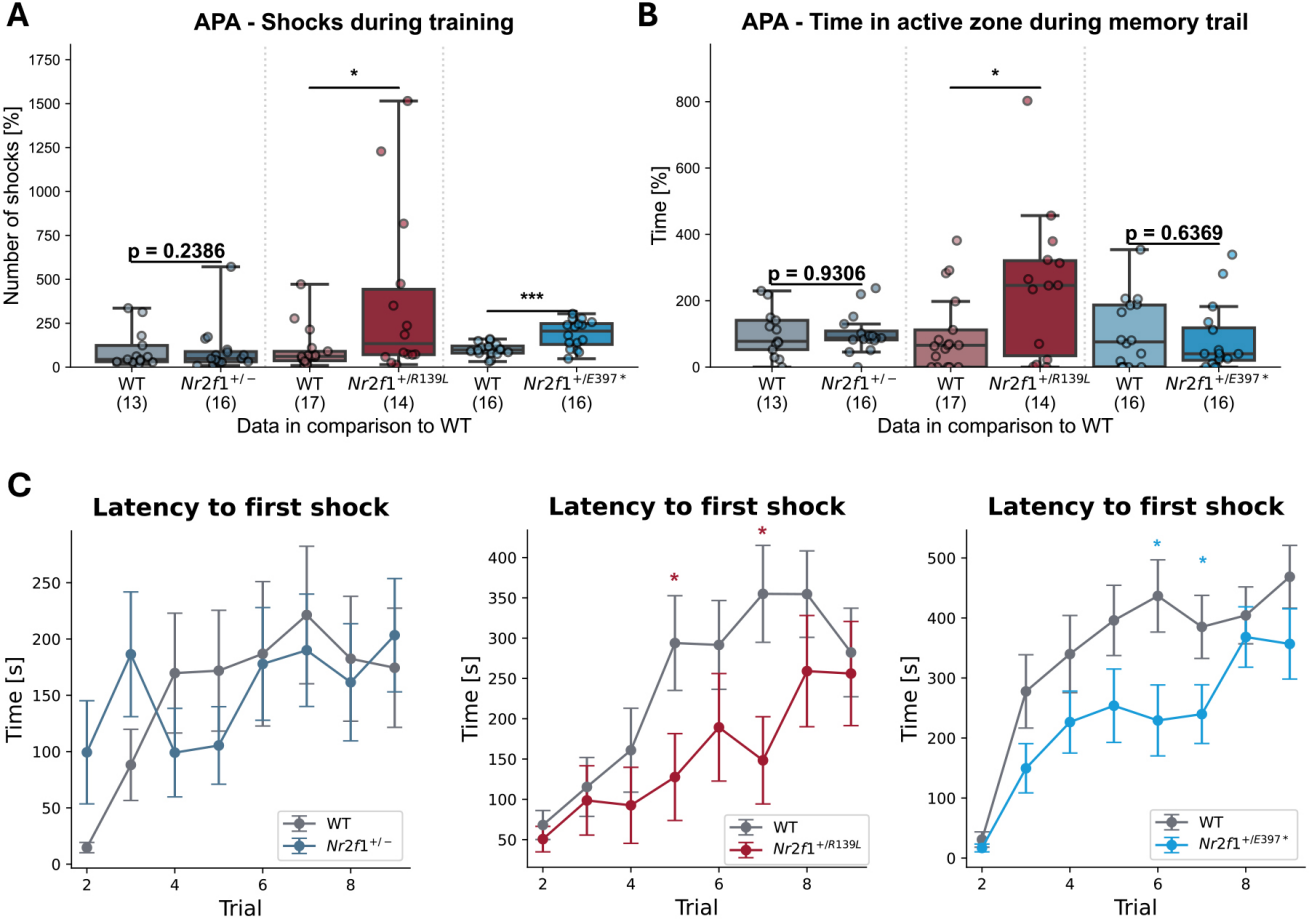
**Learning and spatial memory in the active place avoidance (APA) test.** (A) *Nr2f1^+/R139L^* and Nr2f1^+/E397*^ mice receive more cumulative shocks than their respective WT littermates during the eight training trials of the APA test. (B) *Nr2f1^+/R139L^* mice spend more time in the inactive shock zone during the recall trial, 24 h after the training, than WT littermates. Datapoints for each line are shown, normalized to the respective WT mean. Box plots represent the median and 25-75th percentiles; whiskers indicate the range of the data. (C) Descriptive figures show the learning process of the variant-carrying mice in comparison to that of the respective WT littermates. Bars represent s.e.m. *P≤0.05, ***P≤0.001; Student's *t*-test.

To test the spatial learning and memory properties of our Nr2f1 mutant mice, we used the active place avoidance (APA) test, with the primary readout being the total number of shocks received over eight training trials. Both *Nr2f1^+/R139L^* and Nr2f1^+/E397*^ mice received significantly more shocks (484% and 188% increase in number, respectively) and spent more time (652% and 197% increase, respectively) in the shock zone compared to their WT littermates during training (Fig. 6A; Table S6). To evaluate memory retention, animals were reintroduced to the arena 24 h later for a memory trial in which the shock zone was turned off. We assessed their ability to recall the location of the previously active shock zone. One parameter, the latency to the first entry into the previously shock-associated active zone, was on average 13% shorter for *Nr2f1^+/−^* mice, 26% shorter for *Nr2f1^+/R139L^* and 15% longer for Nr2f1^+/E397*^ mice, compared to that for WT littermate controls; however, these differences were not statistically significant (Table S6). Notably, *Nr2f1^+/R139L^* mice spent significantly more time in the previously active zone during the memory trial, suggesting a possible deficit in spatial memory recall (Fig. 6B).

To illustrate the learning process, Fig. 6C depicts the average latency (in seconds) for animals of each line to first enter the shock zone. During the eight learning trials, animals gradually associate the shock zone with pain and learn to avoid it. As a result, latency is expected to increase over the initial trials. The data reveal that *Nr2f1^+/R139L^* and Nr2f1^+/E397*^ mice exhibit impairments in this learning process. Although these mice eventually learn how to identify and avoid the shock zone effectively, they require more trials to do so. *Nr2f1^+/−^* mice did not show alterations in the APA test.

These results correlate different patient-specific *Nr2f1* mutations with deficits in learning and spatial memory recall, with DBD- and LBD-mutated mouse models displaying the most significant anomalies.

### *Nr2f1^+/R139L^* mice exhibit the most severe phenotype among BBSOAS mouse models analyzed

**Fig. 7.**
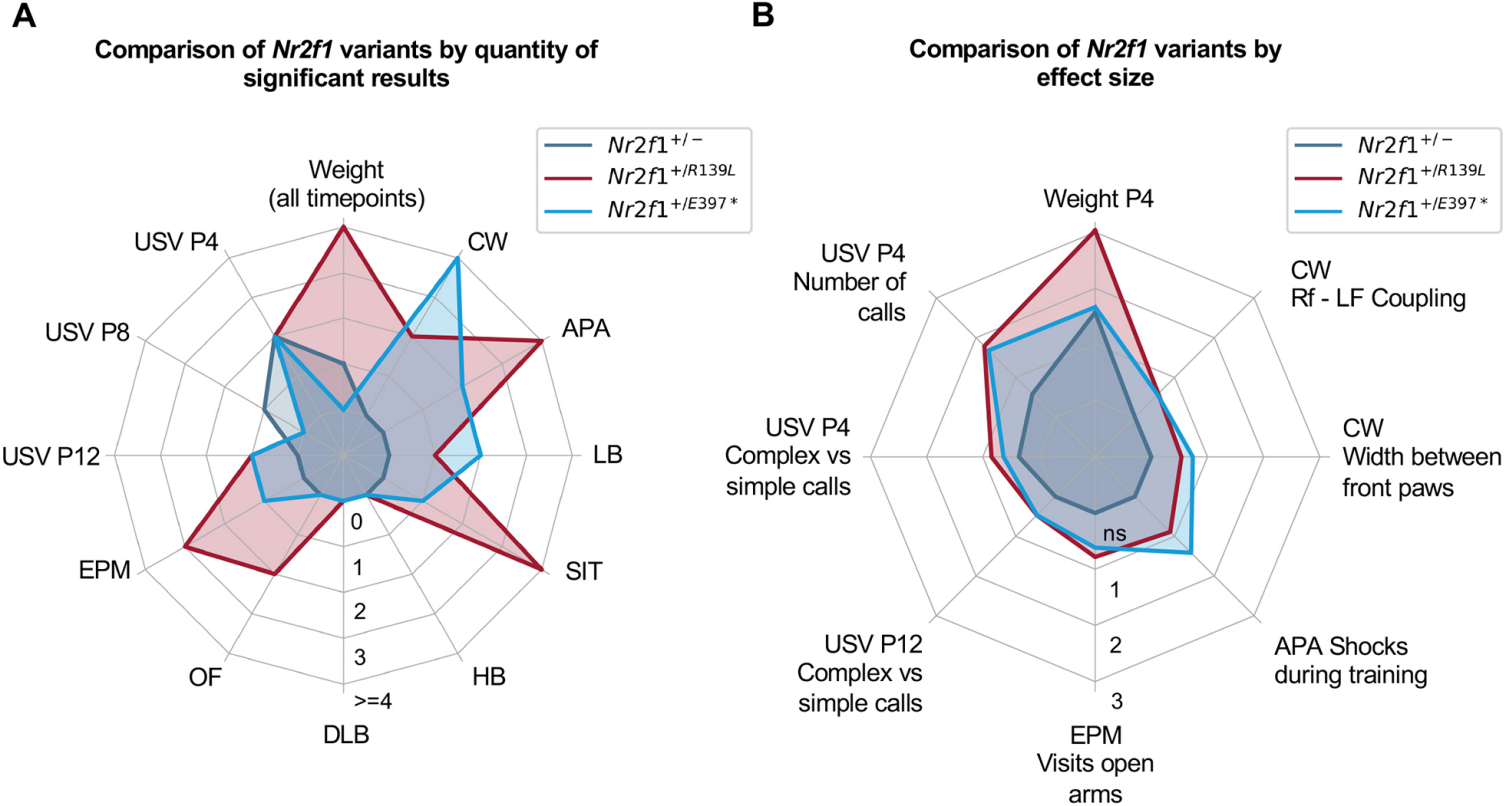
**Quantitative and qualitative analysis of the phenotypic differences between the mouse lines.** (A) Illustration of the number of significant results (as evaluated by Student's *t*-test) for each experiment and mouse model. Because the tests vary in the number of measured parameters, and to maintain clarity and comparability, the radial scale was clipped at 4. For a detailed breakdown, see Table S4. (B) The effect of each mouse model for parameters significant across more than one mouse model was compared using Cohen's *d* (see Table S5). If the parameter was only significant in two of the three models, the non-significant value for the third line was set to 0 (not significant). Cohen's *d* increases with the effect size, with values above 0.8 indicating a strong effect. APA, active place avoidance; CW, CatWalk XT; DLB, dark-light box; EPM, elevated plus maze; HB, hole board; LB, Laboratory Animal Behavior Observation Registration and Analysis System (LABORAS); OF, open field; SIT, social interaction test; USV, ultrasonic vocalization.

Among the three mouse models analyzed in this study, *Nr2f1^+/R139L^* mice displayed the most severe phenotype. Owing to differences in experimental cohorts, timing and potential genetic drift, direct cross-line comparisons were not feasible. Therefore, we performed line-specific analyses, systematically comparing each mutant line to its respective WT littermates. To estimate the overall phenotypic impact of each variant, we quantified the total number of significantly altered parameters. *Nr2f1^+/R139L^* mice exhibited the highest number of significantly altered parameters (24), followed by Nr2f1^+/E397*^ mice (17) and *Nr2f1^+/−^* mice (three) (Fig. 7A; Table S4).

For parameters that were significantly altered in more than one line, we compared the effect sizes using Cohen's *d*. According to Cohen's classifications, effect sizes above 0.2 are considered a mild effect, values above 0.5 are moderate, and values exceeding 0.8 represent a strong effect. The average *d* values were 0.6810, 1.1035 and 1.0054 for *Nr2f1^+/−^*, *Nr2f1^+/R139L^* and Nr2f1^+/E397*^ mice, respectively (Fig. 7B; Table S5), further supporting the conclusion that the *R139L* variant exerts the strongest phenotypic effect.

## DISCUSSION

To date, our pathophysiological understanding of the diverse *NR2F1* variants underlying BBSOAS remains incomplete, limiting the development of targeted therapies. Existing mouse models, such as the heterozygous *Nr2f1* KO mice, fail to fully capture the range of genetic alterations reported in human patients and do not sufficiently recapitulate the broad spectrum of behavioral alterations characteristic of the syndrome (Chen et al., 2020; Flore et al., 2017; Contesse et al., 2019; Parisot et al., 2017; Tomassy et al., 2010). This highlights a critical need for novel mouse models with greater construct validity that more accurately phenocopy both the clinical manifestations and the proposed genotype-phenotype correlations.

In this study, we established two new mouse models carrying patient-specific *NR2F1* variants, selected to represent the more severe end of the phenotypic spectrum observed in BBSOAS. All mouse models assessed in behavioral tasks were bred, maintained and tested under standardized conditions in the same facility. To ensure consistency and comparability, behavioral testing was performed using identical protocols by the same investigator. Our findings demonstrate that all three models exhibit robust, multifaceted phenotypes, with clear genotype-dependent differences. Consistent with previous studies, *Nr2f1*^+/−^ mice displayed only mild behavioral changes and minor brain abnormalities. In contrast, and in line with BBSOAS clinical data, the patient-specific point mutation models (*Nr2f1^+/R139L^* and Nr2f1^+/E397*^ mice) showed more pronounced and complex phenotypes, supporting the hypothesis of a dominant-negative effect associated with missense or nonsense variants in patients.

One example of the dominant-negative effect associated with the DBD variant is reflected in the brain morphology across the models. Mice harboring a DBD variant exhibit several significant anomalies across different brain regions, whereas heterozygous KO mice show alterations limited to thinning of the corpus callosum. However, previous studies have additionally reported hippocampal volume loss in both tissue-specific and constitutive heterozygous *Nr2f1* KO mice (Flore et al., 2017; Chen et al., 2020). The hippocampus is of particular interest, given its relevance to intellectual disability and potential improvements in long-term memory reported in individuals with BBSOAS. Patients also show structural abnormalities such as medial temporal lobe dysgyria (Desai et al., 2023; Pinter et al., 2001; Bertacchi et al., 2020). However, direct comparison with human MRI data remains challenging owing to interspecies differences in brain development, as well as methodological limitations.

Although we did not observe reduced hippocampal volume in the *Nr2f1^+/−^* model, both the *Nr2f1^+/R139L^* and Nr2f1^+/E397*^ mice exhibited marked reductions at various postnatal stages. A consistently observed feature across models, and one that aligns with neuroimaging findings in patients with BBSOAS, is the thinning of the corpus callosum, a hallmark phenotype in the disorder (Chen et al., 2016; Desai et al., 2023; Bertacchi et al., 2020). Notably, our analysis has revealed a previously undescribed phenotype in the point mutation models: a significant enlargement of the lateral ventricles. This novel finding merits further investigation to determine whether it reflects underlying brain atrophy, developmental hypoplasia or disrupted cerebrospinal fluid dynamics.

In parallel with the observed neuroanatomical alterations, we identified several behavioral abnormalities across the three *Nr2f1* mouse models. Our behavioral assessments focused particularly on autism-like features in rodents (USVs and SIT). Notably, all models exhibited a robust reduction in USVs at P4, with the strongest impairments observed in *Nr2f1^+/R139L^* and Nr2f1^+/E397*^ mice, and a milder deviation in *Nr2f1^+/−^* mice. USVs are considered innate behaviors in rodents, and a reduced number of calls is a well-established hallmark in mouse models with autism-like phenotypes, making this parameter a robust and intuitive readout for modeling BBSOAS-related social impairments (Kikusui et al., 2011; Ey et al., 2013). Consistent with clinical data indicating that social deficits in BBSOAS are not confined to a particular developmental window, *Nr2f1^+/R139L^* and Nr2f1^+/E397*^ mice exhibited persistent qualitative alterations in USV patterns at later developmental stages, alongside anomalies in social behavior as assessed by the SIT at P28/P29. Although Nr2f1^+/E397*^ mice displayed a comparatively weaker phenotype in the SIT, they still demonstrated reduced social interest in line with the findings from *Nr2f1^+/R139L^* mice. The reasons for the mild SIT phenotype observed in Nr2f1^+/E397*^ mice and the lack of detectable abnormalities in *Nr2f1^+/−^* mice have not been thoroughly investigated, but could be influenced by several confounding and systemic factors, such as affective state of the animals, experimental setup, limited sample size and the age of the mice, all of which can obscure smaller behavioral effects. Interestingly, our findings in the *Nr2f1^+/R139L^* model are consistent with the results reported by Zhang et al. (2020), who observed similar social impairments in mice carrying a variant in the DBD.

The hyperactive phenotype observed in our experiments, characterized by increased total distance traveled, is particularly notable in light of the subtle yet distinct motor dysfunctions identified through CW analysis. Gait alterations, such as changes in stride length and paw placement, suggest impaired coordination that mirrors clinical findings in patients with BBSOAS, in whom hyperactivity often coexists with motor delays or hypotonia. These results imply that hyperactivity in the mouse models does not solely reflect behavioral changes but could also stem from underlying neuromotor dysfunction, highlighting the value of combining behavioral and gait assessments to capture the full spectrum of BBSOAS-related phenotypes.

To date, four studies have evaluated motor function in different *Nr2f1* models (Tomassy et al., 2010; Contesse et al., 2019; Chen et al., 2020; Zhang et al., 2020). Loss of *Nr2f1* in neocortical and hippocampal neurons led to deficits in fine motor coordination tasks (e.g. skilled-reaching and adhesive-removal task), whereas general motor performances (rotarod and wire hang test) remained unaffected (Tomassy et al., 2010; Contesse et al., 2019). Similarly, Chen et al. (2020) reported no major motor deficits in constitutive heterozygous *Nr2f1* KO mice. Furthermore, Zhang et al. (2020) found no abnormalities in rotarod in their DBD model, although only male mice were assessed. Based on these findings and our own experience with the three mouse models, we did not anticipate a pronounced motor phenotype. However, given previous evidence of *Nr2f1*-related prefrontal/motor area defects, we employed the CW system to detect any subtle motor coordination deficits (Tomassy et al., 2010; Armentano et al., 2007). This approach revealed a more nuanced motor phenotype, with significant gait alterations in *Nr2f1^+/R139L^* and Nr2f1^+/E397*^ mice. Notably, the same models consistently showed increased front paw width, a hallmark often reported in other models of coordination deficits (Scarrott et al., 2023). In addition, a wider stance suggests impairments in balance and coordination. Because front paw width typically correlates with body weight, this effect is expected to be more pronounced in lighter mice, which would show a narrower stance (Pitzer et al., 2021). However, despite their lower body weight, variant-carrying mice show an increased front paw width. The disproportionate stance suggests a fundamental disruption in balance and motor control.

The mutation-dependent effects observed in paw coupling, particularly pronounced in *Nr2f1^+/R139L^* mice, underscore deficits in a coordination task closely linked to fine motor functions. These findings support the hypothesis that Nr2f1 plays a greater role in fine motor control than in gross motor performance (Tomassy et al., 2010). Overall, the lack of significant impairments in general motor performance aligns with both our direct observations and previous reports, reinforcing the idea that Nr2f1-related dysfunction primarily affects coordination rather than basic motor ability.

BBSOAS is associated with a range of neurological and behavioral features, among which intellectual disability is one of the most impactful symptoms, significantly affecting the ability of patients to engage in daily life. Although nearly all individuals with BBSOAS present some degree of intellectual disability, its severity varies widely, ranging from mild to severe (Bertacchi et al., 2022). This phenotypic variability presents challenges for modeling in animals, yet it also underscores the value of cognitive testing in mouse models as a means to dissect genotype-phenotype correlations.

Previous studies have demonstrated spatial learning and memory deficits in *Nr2f1* neocortical- and hippocampal-specific KO models using the Morris water maze (Flore et al., 2017). In line with the improved long-term memory observed in some patients, Chen et al. (2020) also observed altered fear memory in constitutive heterozygous *Nr2f1* KO mice using the fear conditioning test (Chen et al., 2020; Rech et al., 2020). To build on these findings, we conducted the APA test. In accordance with earlier reports, *Nr2f1^+/−^* mice showed no significant changes in learning and memory. However, both *Nr2f1^+/R139L^* and Nr2f1^+/E397*^ mice displayed marked learning impairments.

A potential explanation for the absence of learning and memory deficits in *Nr2f1^+/−^* mice may be their reliance on non-spatial strategies. Rather than encoding specific spatial locations, these mice might compensate for hippocampal dysfunction, particularly deficits in allocentric spatial processing, by adopting alternative strategies such as memorizing distances or movement patterns (DiMattia and Kesner, 1988; Flore et al., 2017). Although the heterozygous KO mouse is an established model for BBSOAS, its behavioral phenotype remains mild, potentially owing to the greater sensitivity of human cognitive assessments in detecting subtle impairments not captured by standard rodent paradigms.

Patients with BBSOAS are frequently described as having remarkably good long-term memory (C.P.S., unpublished observations). Although Chen et al. (2020) reported enhanced long-term memory in a *Nr2f1* variant model based on contextual fear conditioning, our APA memory trials revealed an opposing trend. This discrepancy may be attributable to impairments during the learning phase, which could impact subsequent memory performance. However, as illustrated in Fig. 5C, the animals ultimately learn the location of the active zone, indicating that learning is delayed rather than absent. Further investigations are required to delineate the specific memory-related phenotypes associated with *Nr2f1* variants.

Nevertheless, the novel mouse models presented in this study offer a good representation of the key features of BBSOAS symptoms, including autism-like behaviors, hyperactivity, fine motor dysfunction and intellectual disability. Importantly, our results provide the first experimental evidence that pathogenic point mutations in the DBD (and to a lesser extent in the LBD) lead to more severe phenotypes than the heterozygous KO. When compared to the symptom severity index proposed by Bertacchi et al. (2022) for the different classes of *NR2F1* variants (deletions, DBD and truncation variants), our findings align closely with clinical observations, with heterozygous variants in the DBD causing the most pronounced phenotype followed by truncations (Nr2f1^+/E397*^ in the LBD) and deletions. These results support the notion of a genotype-phenotype correlation and suggest a dominant-negative mechanism, which needs to be further understood. Bertacchi et al. (2022) proposed functional obligatory dimerization as a possible explanation, but neomorphic effects altering Nr2f1 DNA-binding specificity may also contribute and need to be investigated. Furthermore, it is important to note that only 63% of the known patients fall into one of the three main variant categories (16% deletions, 35% DBD variants and 12% truncations) (Bertacchi et al., 2022). Owing to practical constraints, rarer genotypes, such as frameshift variants (8%), were not represented in this study. Moreover, although we analyzed variants that we consider broadly representative, most variants occur *de novo*. Although clinical data suggest that variants can be categorized by locus and type, the absence of recurrent hotspot variants limits our ability to generalize findings. In addition, further investigation into the potential contribution of visual impairment observed in affected individuals to behavioral abnormalities such as hyperactivity, anxiety or motor dysfunction will be essential for a more precise characterization and understanding of the phenotype.

In summary, the three mouse models presented here faithfully replicate key aspects of disease-relevant features of BBSOAS and provide a valuable platform for investigating disease mechanisms. In particular, the novel *Nr2f1^+/R139L^* and Nr2f1^+/E397*^ models offer new opportunities for investigating the underlying pathophysiology of more severe cases of BBSOAS. The pronounced phenotypes observed in these models also open up new therapeutic avenues for symptom mitigation. Reliable models are essential for exploring therapeutic strategies and, based on the strong dominant-negative effect observed in *Nr2f1^+/R139L^* mice, we have initiated therapeutic studies using antisense oligonucleotide-based approaches to selectively downregulate the pathogenic *R139L* allele. In addition, these models are relevant beyond the scope of BBSOAS, providing the scientific community with novel tools to study autism-like behaviors, coordination deficits and broader neurodevelopmental abnormalities.

In conclusion, the presented models provide a robust platform for dissecting BBSOAS pathophysiology and improving our understanding of genotype-phenotype correlations. Further studies, including complementary behavioral phenotyping and therapeutic approaches, are ongoing. Addressing these challenges will ultimately require a coordinated effort among teams specializing in neurodevelopmental disorders, and we are open to collaborating with others. We anticipate that our work will significantly enhance the understanding of BBSOAS and serve as a valuable resource for the NR2F1 research community.

## MATERIALS AND METHODS

### Animals

Mice were maintained in a specific-pathogen-free facility at the Interfaculty Biomedical Facility (IBF) of Heidelberg University and at the Centre d'Exploration Fonctionnelle Pré-clinique of the Institute of Biology Valrose, University Côte d'Azur, Nice, France, on a 12-h light/dark cycle. They had *ad libitum* access to food and water. Environmental conditions were controlled at 50-60% humidity and 22°C (±2°C).

The behavioral experiments were performed at the Interdisciplinary Neurobehavioral Core in Heidelberg by the same experimenter, who was unaware of mouse genotype. All animals started on the same behavioral timeline. This was done to ensure that all animals had the same experimental experience at all stages. Testing was performed in two cohorts, consisting of 66 and 142 animals, respectively. Animals carrying a variant were tested alongside their wild-type littermates. Genotyping was performed after the USV test, and the cohorts were subsequently reduced to the target group size of ∼14 animals per group by randomly excluding surplus litters or littermates (see Table S7). The tests were conducted during the light phase and in strict compliance with national and international guidelines for the Care and Use of Laboratory Animals. The experimenter ensured that background noises during testing were kept to a minimum. The study was approved by the Regional Council Regierungspräsidium Karlsruhe, Germany (G-172/21, G-177/21 and G-252/19), and by the local ethical committee (CIEPAL Azur 28 NCE/2024-976) and the Ministry for Higher Education and Research, France.

We tested three genetic mouse lines carrying patient-specific *NR2F1* variants – *Nr2f1^+/−^*, *Nr2f1^+/R139L^* and Nr2f1^+/E397^* – all maintained on a C57BL/6J background (Armentano et al., 2006). Littermates from each respective mouse line served as wild-type controls. Each model represents a distinct patient subgroup, and both male and female mice were tested in balanced proportions and showed similar behavior (Fig. S1 and Table S7). For details and genotyping information, refer to Table S1.

### Generation of the *Nr2f1^+/−^* mouse model

*Nr2f1^+/−^* mouse embryos were obtained via the European Mouse Mutant Archive (EMMA-international strain name B6.129S2-Nr2f1^tm1Mist^/Cnrm), and the colony at the IBF was established via embryo transfer. For details on the generation of this mutation, see Armentano et al. (2006).

### Generation of the *Nr2f1^+/R139L^* mouse model

The *Nr2f1^+/R139L^* mouse line was generated at the Baylor College of Medicine in Houston, TX, USA, by the Genetically Engineered Rodent Models Core using CRISPR/Cas9-mediated genome editing. The mutation was introduced at the end of the first exon of *Nr2f1*. The mutation replaced the CGC codon (arginine) with a CTC codon, resulting in the substitution of arginine by leucine (R139L) (see Fig. S6 for CRISPR/Cas9 design). To facilitate genotyping, donor DNA was designed to introduce the desired mutation along with silent mutations, and sequencing primers were developed targeting the mutated region. The Cas9 protein and the single-guide RNA (sgRNA), together with the single-stranded oligodeoxynucleotide repair template, were microinjected into fertilized C57BL/6J oocytes. Following microinjection, chimeric founders were generated and subsequently mated with C57BL/6J females to achieve germline transmission of the mutation.

### Generation of the Nr2f1^+/E397*^ mouse model

The E397* mouse line was generated at PHENOMIN-iCS (Institut Clinique de la Souris, Illkirch, France) as part of the PHENOMIN-2020 call for proposals, with funding provided by PHENOMIN. Three successive point mutations (CGA>ATG) were introduced at the beginning of exon 3 of the *Nr2f1* gene, targeting nucleotide position (1188 to 1190). Two of the mutations converted the GAA codon (glutamic acid) into a TGA stop codon, resulting in a premature termination at amino acid residue 397 (E397*) (see Fig. S7 for CRISPR/Cas9 design). The third mutation was silent at the protein level but introduced an NdeI restriction site to facilitate genotyping. Wild-type Cas9 protein and the CRISPR RNA (crRNA)/trans-activating CRISPR RNA (tracRNA) (crRNA sequence 5′-tctcggatgagagtttcgat-3′ score 88 according to https://crispor.gi.ucsc.edu/) and a single-stranded repair template containing the desired mutations, were electroporated into fertilized C57BL/6N oocytes. Two mosaic founders were obtained and mated with C57BL/6N females to achieve germline transmission of the mutation. Prior to experiments, the line was backcrossed (eight to ten generations) into the C57BL/6J background. For primer details see Table S1. The amplified products were digested with Nde1 (30 min at 37°C) to discriminate the wild-type allele from the mutant.

### Real time qRT-PCR

E14.5 mouse embryo brains were dissected, cut in half and frozen at −80°C. Total RNA was isolated with NucleoSpin RNA II columns (Macherey-Nagel, 740902.50). RNA quantity and quality were evaluated with a Nanodrop spectrophotometer (Thermo Fisher Scientific) and agarose gel electrophoresis. For each sample, 200 ng RNA was reverse transcribed using random nonamers (Reverse Transcriptase Core Kit, Eurogentec, qRT-RTCK-03). Quantitative reverse transcription PCR (qRT-PCR) was performed with GoTaq SYBR Green qPCR Mix (Promega, A6001) on a LightCycler^®^ 480 (Roche). cDNA, stored at −20°C, was diluted to ensure 2 ng per reaction. Amplification take-off values were determined using the built-in LightCycler^®^ 480 relative quantification analysis function, and relative expression was calculated with the 2^−ΔΔCt^ method, normalizing to the housekeeping gene β-actin. At least two reactions were assembled per sample/gene analyzed during qRT-PCR amplification (technical replicates). Primer sequences used are listed in Table S1.

### Cresyl Violet (Nissl) staining protocol

Mice were anesthetized using a mixture of Tiletamine-Zolazepam-Xylazine-Buprenorphine (TZXB) and perfused intracardially with phosphate-buffered saline (PBS), followed by 4% paraformaldehyde (PFA) in PBS. Brains were dissected, postfixed in 4% PFA for 24 h at 4°C, and then cryoprotected by sequential immersion in sucrose-PBS solutions (10% sucrose for 12 h, then 25% for 20 h at 4°C) before being embedded in Cryomatrix (Epredia, 6769006) and stored at −80°C. Coronal brain sections (40 µm) were cut using a Leica cryostat and collected as free-floating sections in 12-well plates containing PBS with 0.05% sodium azide. Brain sections were then mounted and air dried for 12 h at RT. Slides were briefly post-fixed in 4% PFA for 10 min at RT, followed by three washes in PBS (5 min each). Sections were then defatted in a 1:1 acetone: ethanol solution for 10 min at RT and equilibrated in MilliQ water for 2 min. Staining was performed by incubating slides for 1 h at 37°C in 0.1% Cresyl Violet Acetate (Sigma-Aldrich, C5042) prepared in 0.2% acetic acid in water. After staining, slides were rinsed in 96% ethanol, quickly dehydrated in 100% ethanol and cleared in xylene for 5 min. Finally, sections were coverslipped using VectaMount mounting medium (VectorLabs, H-5000).

### Brain MRI

Brain MRI was performed in adult mice under isoflurane anesthesia (induction, 4%; maintenance, 1.5-2%) using a 3T scanner. T2-weighted images were acquired with a RARE sequence (repetition time, 3350 ms; echo time, 48 ms) to assess brain morphology. Images were acquired at a resolution of 0.104 mm×0.104 mm with an image matrix of 192×192 and a field of view of 20 mm×20 mm. Body temperature was maintained at 37°C, and respiration was monitored throughout the procedure. Image analysis was conducted using Horos, 3D Slicer and IMARIS to assess volumetric and structural differences. In brief, brains and ventricles were manually segmented based on T2 hyperintense signal and exported as binary labelmaps. A surface mesh was generated using ITKSnap and imported to IMARIS for 3D visualization and rendering.

### Neonatal USV

At P4, P8 and P12, pups were randomly selected, separated from their mother and littermates, and placed in a sound-attenuating chamber (42 cm×42 cm×42 cm) with 350 lx illumination. All sounds emitted by the pups were recorded for 5 min. Following the session, pups were immediately returned to their home cages.

USVs were recorded using an UltraSoundGate condenser microphone (Avisoft Bioacoustics, CM16/CMPA) positioned 30 cm above the testing arena. For analysis, a custom-developed software was utilized, employing entropy-based thresholding and a convolutional neural network (Herdt et al., 2024). The software enables the detection and classification of individual USVs into predefined categories. To validate the accuracy of the analysis, randomly selected samples were manually verified using SASLabPro software (Avisoft Bioacoustics). Calls were detected with 94.9% recall and 99.3% precision (Herdt et al., 2024). The subsequent classification achieved an accuracy of 86.8% (Herdt et al., 2024).

### EPM

At P21, mice were placed in the center of an arena. Voluntary movements were automatically tracked (Sygnis Tracker, custom-made) for 10 min. The brightness was controlled at 210 lx in the center, 200 lx in the open arms and 170 lx in the closed arms (Ayala et al., 2010).

### OF

The central area brightness was set to 250 lx. At P22, mice were placed in alternating corners of an arena, and their spontaneous movement was recorded (Sygnis Tracker) for 10 min.

### DLB

At P24, mice were placed at the threshold between the light and the dark compartment, and their movements were automatically tracked (Sygnis Tracker) for 10 min. Brightness levels were set at 300 lx in the light compartment and 2 lx in the dark compartment (Bourin and Hascoët, 2003).

### HB

At P26, mice were placed on a board for a 10-min session, and head dips were manually recorded. Tests were conducted at 170 lx brightness.

### SIT

At P28 or P29, mice were placed in the arena in three consecutive trials. The arena was illuminated at 160 lx. During the first trial, the test animal explored the three chambers. In the second trial, a same-sex littermate was placed inside a pencil cup in one of the side chambers. In the third trial, a novel intruder mouse, a same-sex adult CD-1 mouse, was introduced into the other chamber. Each trial lasted 5 min, with the test animal initially placed in the middle chamber. A 15-min interval was maintained between trials. The time spent and distance traveled in each chamber were automatically recorded.

### LABORAS

The LABORAS platform is a home-cage monitoring system. We measured behavioral parameters between P31 and P35, including locomotion, grooming, rearing and climbing. The testing environment during the light phase was maintained at 160 lx. Mice were individually housed in LABORAS cages for 24 h with *ad libitum* access to food and water. Behavior was tracked automatically via the LABORAS software.

### CW

The CW gait analysis system (Noldus, Wageningen, The Netherlands) assessed locomotion at P36, including step sequences, speed and paw coordination. Mice were placed in a 1 m long, red-light illuminated corridor (10 lx). Based on the literature, no training was done (Deumens et al., 2007). Testing continued until three recordings with a run variation below 30% were obtained. For analysis, both the standard parameters provided by the CW system and a machine learning model were used. The computerized gait analysis examines a comprehensive array of 317 parameters. To ensure a focused and interpretable analysis, we only examined specific parameters, e.g. gait speed or the width between the front paws. Additionally, to address the complexity inherent in the CW dataset, we employed a novel machine-learning algorithm developed by Timotius et al. (2021). The machine learning model integrates key CW parameters into a composite index representing overall locomotor performance.

For the analysis of individual parameters, we prioritized a subset of commonly used parameters, selected for their established relevance and intuitive interpretability (Heinzel et al., 2020; Timotius et al., 2021; Machado et al., 2015). Notably, 90% of the 317 parameters assessed by the CW system are speed dependent (Heinzel et al., 2020). However, because none of the mouse models exhibited significant differences in speed (Fig. S4), we performed a motor function deficit-focused comparison without confounding effects from variations in gait speed.

### APA

The APA task was performed on two consecutive days between P40 and P50, based on previously established protocols (Zalucki et al., 2018). The round arena (77 cm in diameter) rotated at 1 rpm. Lighting was set to 100 lx. The EthoVision XT software defined a 60° shock zone and delivered mild electric shocks upon entry to promote avoidance and motivate the animals to exit the zone. Learning was assessed over eight trials with a 20-min break between each, followed by a memory test the next day.

### Data analysis

Outliers (values deviating by more than 3 standard deviations from the mean) were excluded from further analysis. Behavioral data were analyzed using a two-tailed homoscedastic (Student's) *t*-test. All datasets met our prerequisite of having a skewness less than 3. This approach follows recent mathematical recommendations regarding pre-testing, statistical test selection and average sample sizes (Fagerland and Sandvik, 2009; Rasch et al., 2011). All our findings are explorative. Statistical computations were performed using Python (pandas, numpy and scipy.stats libraries). Statistical significance was set at *P*≤0.05 for all analyses.

## Supplementary Material

10.1242/dmm.052426_sup1 Supplementary information
