# Supplementary material for: Models of Bosch-Boonstra-Schaaf optic atrophy syndrome reveal genotype-phenotype correlations in brain structure and behavior
Source: Dis Model Mech. 2025 Sep 22;18(10):dmm052426. doi: 10.1242/dmm.052426 (PMC12505268; doi:10.1242/dmm.052426)
Supplement: Supplementary information [file dmm-18-052426-s1.pdf]

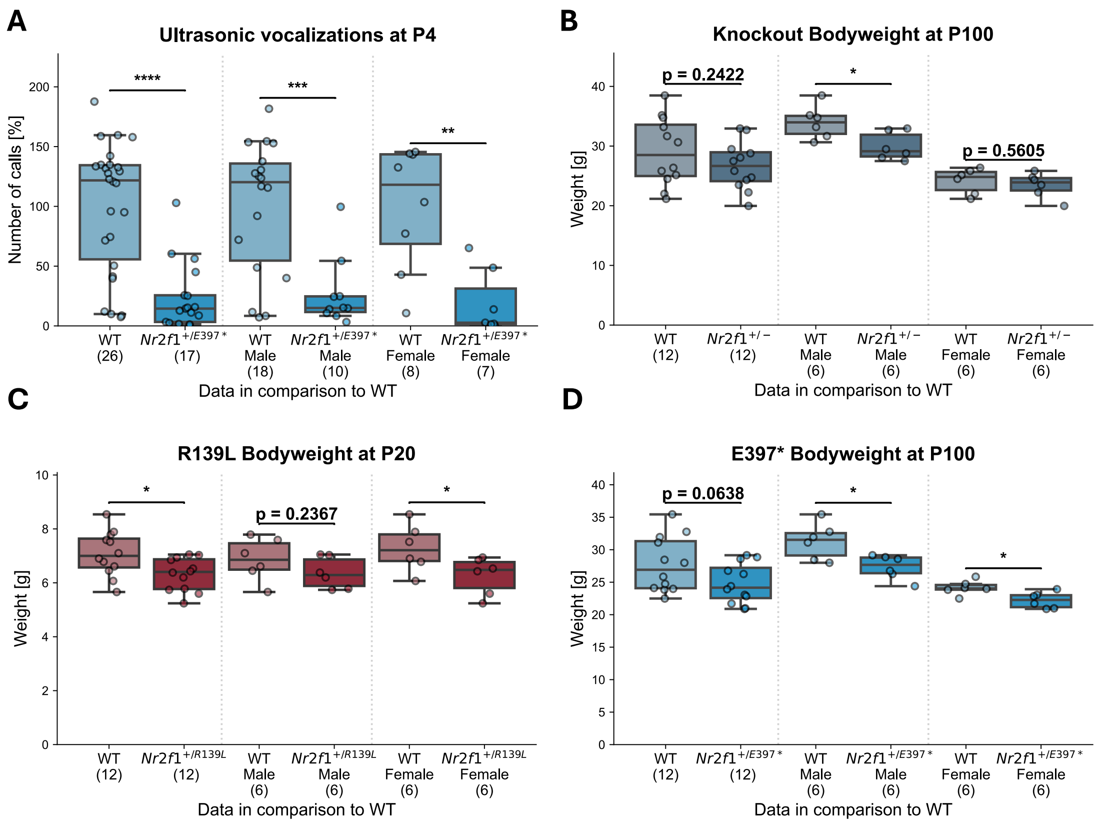

Fig. S1. Sex-specific analysis of selected parameters for distinct time points.

(A) An example of a sex-specific analysis, showing that both sexes show the same behavior. (B) At P100, *Nr2f1*$^{+/-}$ mice show a reduction in body weight for male mice (P=0.0215). (C) At P20, *Nr2f1*$^{+/R139L}$ mice show a reduction in body weight for both sexes (P=0.0176), driven by the females (P=0.0410). (D) At P100, *Nr2f1*$^{+/E397*}$ mice show a reduction in body weight for both males (P=0.0162) and females. Data points for each line are shown, normalized to the respective WT mean. *P ≤ 0.05, **P ≤ 0.01, ***P ≤ 0.001, ****P ≤ 0.0001. Student's t-test.

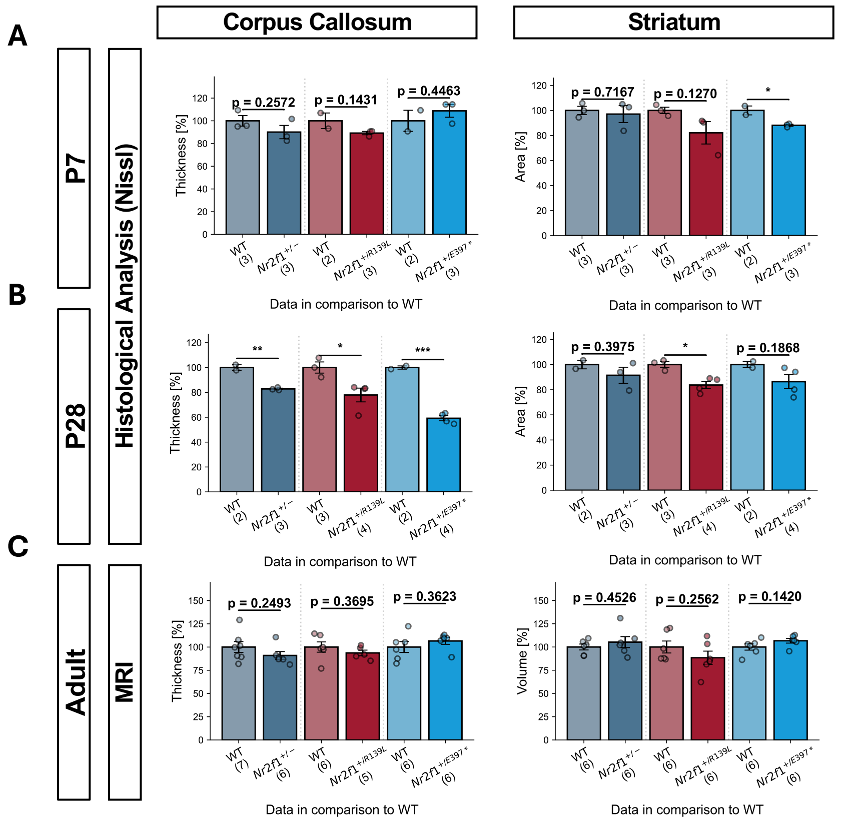

Fig. S2. Corpus Callosum and Striatum in P7, P28, and adult mice.

(A) Quantification of corpus callosum thickness and striatal area in P7 WT, *Nr2f1*$^{+/-}$, *Nr2f1*$^{+/R139L}$, and *Nr2f1*$^{+/E397*}$ brains (Nissl staining). No significant changes in corpus callosum thickness. Slightly reduced striatal area in *Nr2f1*$^{+/E397*}$ mice. (B) Quantification of corpus callosum thickness and striatal area in P28 WT, *Nr2f1*$^{+/-}$, *Nr2f1*$^{+/R139L}$, and *Nr2f1*$^{+/E397*}$ brains (Nissl staining). Significant thinning of the corpus callosum in all mutant lines and reduced striatal area in *Nr2f1*$^{+/R139L}$ mice. (C) Quantification of corpus callosum thickness and striatal volume in adult WT, *Nr2f1*$^{+/-}$, *Nr2f1*$^{+/R139L}$, and *Nr2f1*$^{+/E397*}$ mice (MRI analysis). No significant differences between genotypes. Data normalized to the respective WT mean. *P ≤ 0.05, **P ≤ 0.01, ***P ≤ 0.001; Student’s t-test.

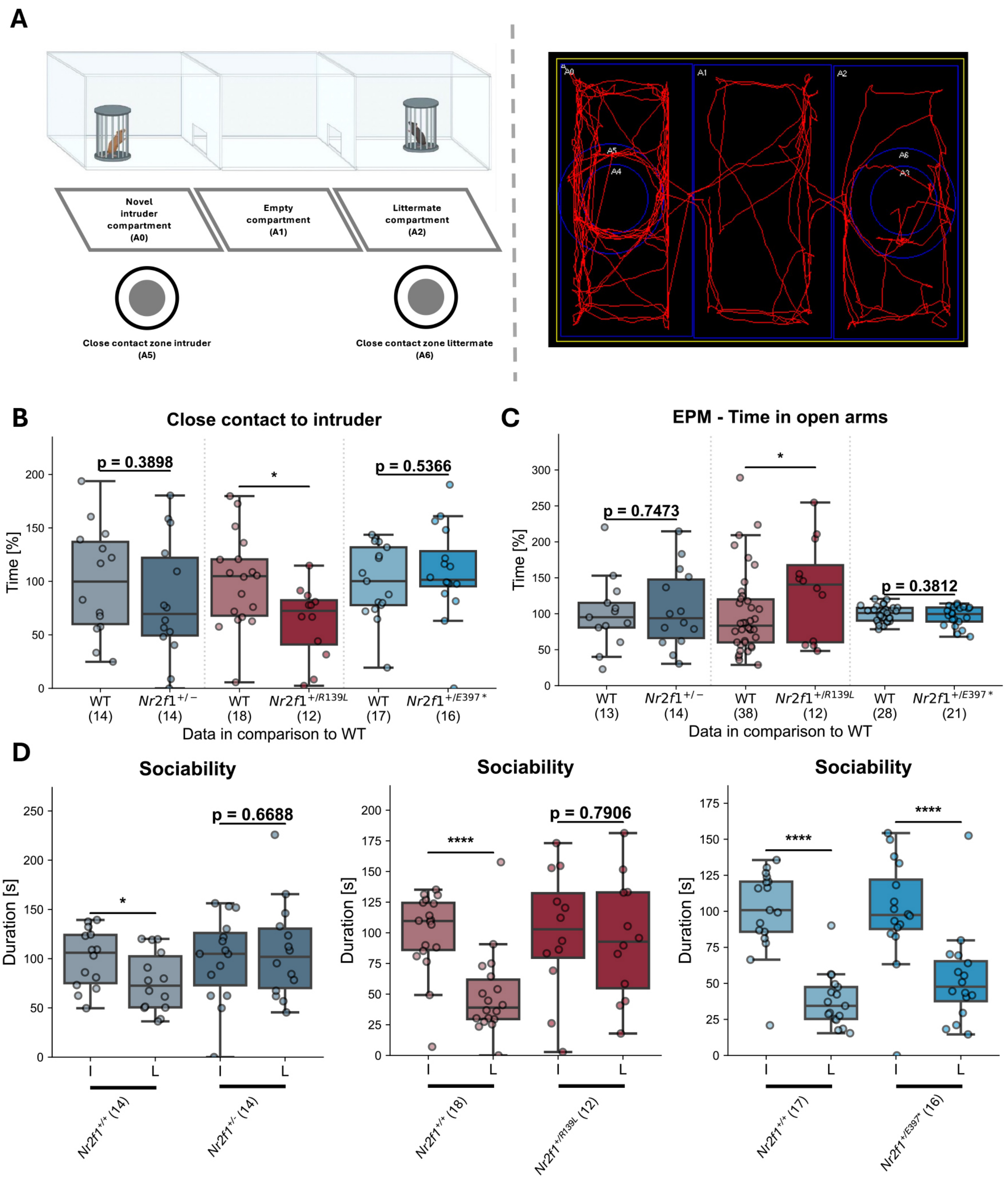

Fig. S3. Alterations in social interaction and elevated plus maze for mice with Nr2f1 variants. (A) An illustration of the SIT setup during trial 3 shows the different areas that were evaluated, as well as an example of a track map from a representative WT animal. (B) *Nr2f1*$^{+/R139L}$ mice spend less time in the close contact zone of the intruder as compared to their respective WT littermates. (C) The *Nr2f1*$^{+/R139L}$ mice spend more time in the open arms of the EPM arena, as compared to their WT littermates. Data points for each line are shown, normalized to the respective WT mean. (D) The graphs compare the sociability of WT and mutant animals for each genetic line. Typically, mice spend more time interacting with the intruder (social novelty) than with their littermates. This behavior is observed in all WT lines. However, animals carrying the R139L variant or a deletion in *Nr2f1* do not exhibit a preference for social novelty, suggesting impaired social interaction abilities. Labels: I - Intruder; L - Littermate. Data points for each line are shown, normalized to the respective WT mean. *P ≤ 0.05, ****P ≤ 0.0001; Student's t-test.

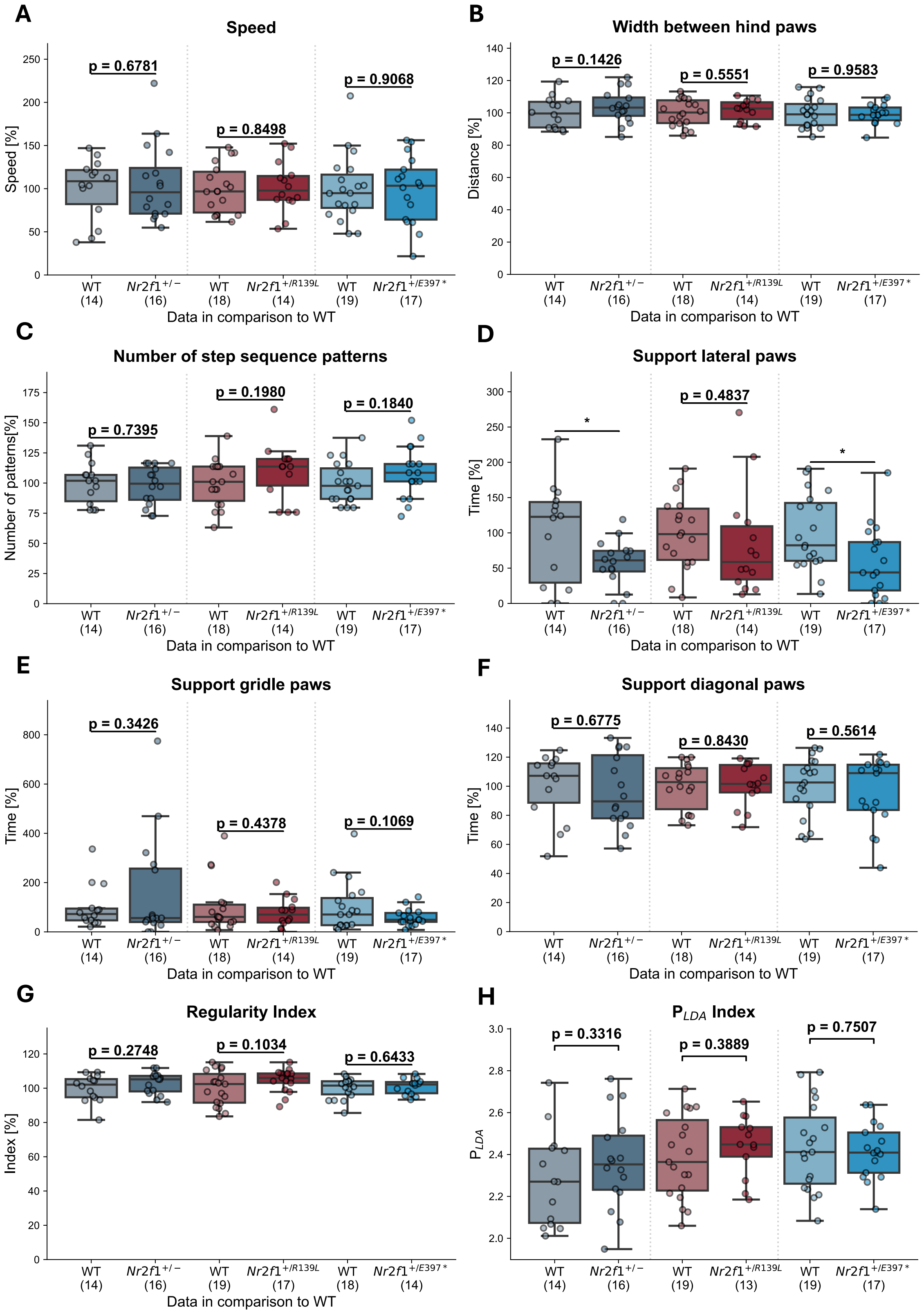

Fig. S4. CatWalk XT-based Motor Function Analysis

(A) The data shows no difference in body speed for all three mouse models. (B) The width between the hind paws in unchanged for the three mouse lines. (C) Mice do not show changes in the number of step sequence patterns in the CW. (D) *Nr2f1*$^{+/-}$ mice and *Nr2f1*$^{+/E397*}$ mice show a decrease in the lateral (e.g. left front, left hind) support. (E) The mice do not show a change in the girdle (e.g. left front, right front) support. (F) The mice do not show a change in the diagonal (e.g. left front, left hind) support. (G) The mice do not show a change in the regularity index, for overall limb coordination. (H) P_{LDA} Idex shows no difference between the lines in overall motor performance. *P ≤ 0.05; Student's t-test.

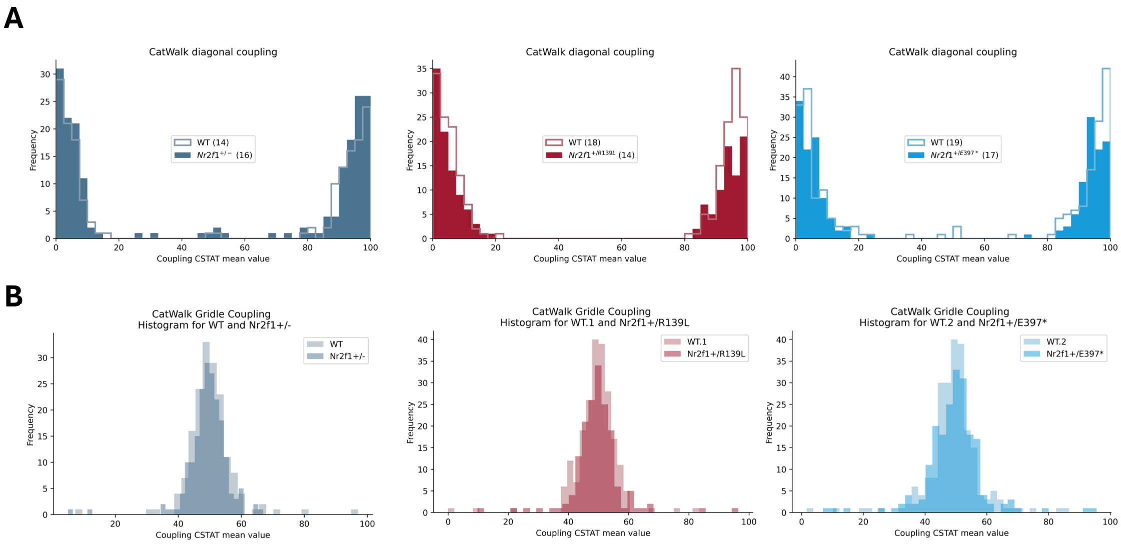

Fig. S5. Interpaw coordination analysis, CatWalk XT.

(A + B) Descriptive illustration of the overall diagonal, and gridle coupling, as measured in CSTAT mean. There are no apparent changes observable.

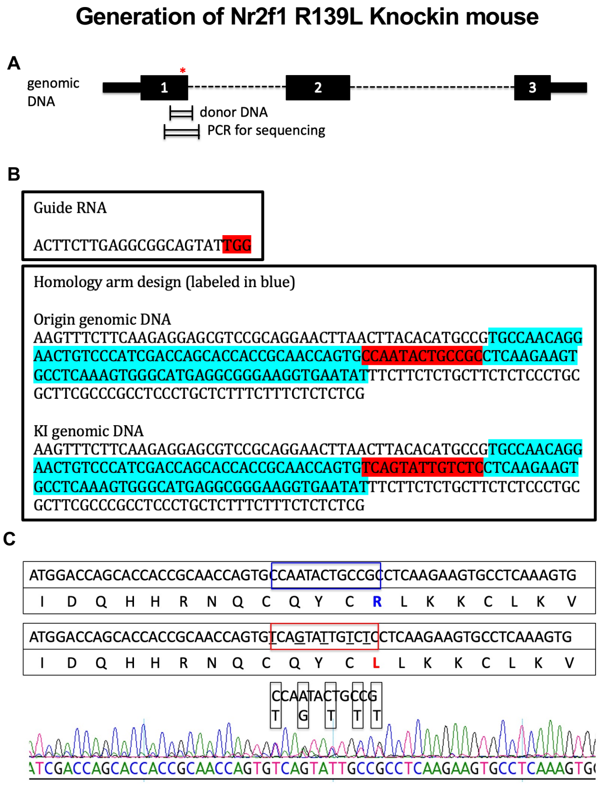

Fig. S6. Illustration of the creation of the *Nr2f1*$^{+/R139L}$ knockin mouse CRISPR/Cas9 design.

(A) Schematic representation of the Nr2f1 genomic locus showing exon 1–3 and the targeting site (red asterisk) within exon 1. The donor DNA and primers used for PCR and sequencing are indicated. (B) Details of CRISPR/Cas9 targeting. The guide RNA sequence (black) and PAM site (red) are shown. The wild-type (origin) genomic sequence and knock-in (KI) genomic sequence are aligned, highlighting the homology arms (blue), the target codon (red), and the introduced mutation.(C) Confirmation of the *Nr2f1*$^{+/R139L}$ mutation at the DNA level. The amino acid change from arginine (R) to leucine (L) is shown in the aligned translated sequences. Sanger sequencing chromatogram confirms the successful introduction of the c.416G>T mutation in the KI allele.

Disease Models & Mechanisms • Supplementary information

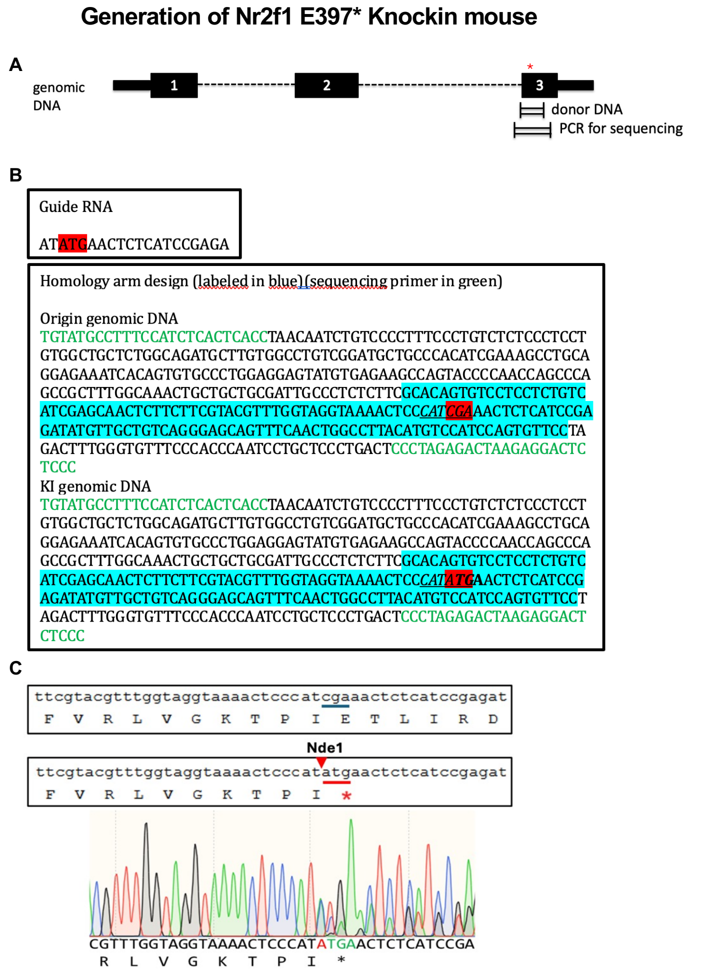

Fig. S7. Illustration of the creation of the *Nr2f1*$^{+/E397*}$ knockin mouse CRISPR/Cas9 design.

(A) Schematic representation of the *Nr2f1* genomic locus showing exon 1–3 and the targeting site (red asterisk) within exon 3. The donor DNA and primers used for PCR and sequencing are indicated. (B) Details of CRISPR/Cas9 targeting.

are aligned, highlighting the homology arms (blue), the target codon (red), and the introduced mutation. (C) Confirmation of the *Nr2f1*$^{+/E397*}$ mutation at the DNA level. The amino acid change from glutamic acid (E) to a premature stop codon (*) is shown in the aligned translated sequences. Sanger sequencing chromatogram confirms the successful introduction of the *c.1189G>T* mutation in the KI allele.

Disease Models & Mechanisms • Supplementary information

Table S1. Primer List.

For the KO line, the amplicon of the null allele is 36bp longer as it contains a loxP site. For the E397* genotyping, subsequently use NdeI to cut the amplicon into a 419 bp long WT band and the 280 bp and 139 bp long fragments of the E397* allele. For the R139L genotyping, there is only amplification in mutated animals as the primers are specific to the mutated sequence. For detailed genotyping protocols, reach out to the corresponding author of this paper. Primer 8-15 were used for real-time qRT-PCR (Fig. 1B-F).

Number	*Name*	*Sequence*
1	genotyping-KO1	5'-CTGCTGTAGGAATCCTGTCTC-3'
2	genotyping-KO2	5'-AATCCTCCTCGGTGAGAGTGG-3'
3	genotyping-KO3	5'-ACATACACAGCCTGGCCTTGC-3'
4	Genotyping E397*-1	5'-TGTATGCCTTTCCATCTCACTCACC-3'
5	Genotyping E397*-2	5'-GGGAGAGTCCTCTTAGTCTCTAGGG-3'
6	Genotyping R139L-1	5'-AGCATCCTCACACACAAGCG-3'
7	Genotyping R139L-2	5'-CCGCAACCAGTGTCAGTATTG-3'
8	Nr2f1 exon3 F	5'-TCCCATCGAAACTCTCATCC-'2
9	Nr2f1 exon3 F	5'-AGTGGGCTGCTCTTGTTCC-3'
10	Nr2f1 R139L WT F	5'-CAACCAGTGCCAATACTGCCGCC-3'
11	Nr2f1 R139L WT R	5'-CAGACAGGTAGCAGTGGCCA-3'
12	Nr2f1 R139L KI F	5'-CAACCAGTGTCAGTATTGTCT-3'
13	Nr2f1 R139L KI R	5'-TAGCCAGACAGGTAGCAGTG-3'
14	Nr2f1 E397* KI F	5'-AAGCCAGTACCCCAACCAG-3'
15	Nr2f1 E397* KI R F	5'-AACATATCTCGGATGAGAGTTCAT-'2

Disease Models & Mechanisms • Supplementary information

Table S2. List of all p-values by Student's t-test for the histological and MRI analyses.
The table shows p-values for histological measures at postnatal days 7 and 28 and for MRI-based volumetric analyses in adult mice. Statistical comparisons were made between wild-type (WT) and mutant (R139L and E397*) groups using Student's t-test. Changes (increase or decrease) compared to WT are indicated where relevant. Outliers (values >3 standard deviations from the group mean) were excluded prior to analysis. For details, see Methods; Histological Analysis, MRI Acquisition and Data Analysis.

Measure	WT vs Null	WT vs R139L	WT vs E397*
**Histology Nissl P7**			
CC thickness before Hipp (µm)	0.8044	0.4360	0.5682
CC thickness after Hipp (µm)	0.1282	0.0585 (decrease)	0.2542
Thickness CC (µm)	0.2572	0.1431	0.4463
Moyenne Anterior hippocampus area (µm^2)	0.0829	0.0044 (decrease)	0.5639
Moyenne posterior hippocampus area (µm^2)	0.1420	0.1981	0.1920
Moyenne Hippocampus total area (mm^2)	0.0632	0.0829	0.3260
Motor cortex thickness (µm)	0.4118	0.7904	0.6028
Somatosensory Cortex Thickness (µm)	0.3073	0.2438	0.2757
Visual Cortex Thickness (µm)	0.3217	0.7385	0.0418 (decrease)
Right ventricule (area µm^2)	0.5393	0.1709	0.1133
Left ventricule (area µm^2)	0.2597	0.0854	0.1397
Total	0.4119	0.0937	0.1245
Right ventricule (perimeter µm)	0.8400	0.1110	0.4706
Left ventricule (perimeter µm)	0.5213	0.0639	0.5167
Ant hippocampale commissure (Area µm^2)	0.6681	0.0254 (decrease)	0.2705
Ant hippocampale commissure (Perimeter µm)	0.8004	0.8456	0.6760
Striatum	0.7167	0.1270	0.0252
Globus Pallidus	0.7495	0.2443	0.0202
**Histology Nissl P28**			
CC thickness before Hipp (µm)	0.0119 (decrease)	0.0301 (decrease)	0.0129 (decrease)
CC thickness after Hipp (µm)	0.5432	0.1465	0.0001 (decrease)
Total Thickness CC (µm)	0.0026 (decrease)	0.0328 (decrease)	0.0002 (decrease)
Moyenne Anterior hippocampus area (µm^2)	0.0811	0.0001 (decrease)	0.3328
Moyenne posterior hippocampus area (µm^2)	0.2270	0.0639	0.0069 (decrease)
Moyenne Hippocampus total area (mm^2)	0.1733	0.0064 (decrease)	0.0466 (decrease)
Motor cortex thickness (µm)	0.3694	0.9382	0.3605
Somatosensory Cortex Thickness (µm)	0.0838	0.4023	0.5209
Visual Cortex Thickness (µm)	0.6170	0.0009 (decrease)	0.7984
Right ventricule (area µm^2)	1 0.3074	0.0187 (increase)	0.0081 (increase)
Left ventricule (area µm^2)	0.3063	0.0172 (increase)	0.0235 (increase)
Total	0.2808	0.0092 (increase)	0.0002 (increase)
Right ventricule (perimeter µm)	–	0.4318	0.1681
Left ventricule (perimeter µm)	–	0.0972	0.2045
Ant hippocampale commissure (Area µm^2)	0.7853	0.0003 (decrease)	0.0824
Ant hippocampale commissure (Perimeter µm)	0.5418	0.6106	0.1326
Striatum	0.3975	0.0100	0.1868
Globus Pallidus	0.4633	0.8861	0.7359
**MRI Adults**			
Striatum	0.4564	0.2562	0.1420
Hypothalamus	0.3818	0.8412	0.8699
Thalamus	0.3006	0.0479 (decrease)	0.7566
Hippocampus	0.3231	0.0043 (decrease)	0.9471
Cerebellum	0.2320	0.3113	0.8942
Corpus Callosum	0.2493	0.3695	0.3623
Lateral Ventricles	0.4966	0.0163 (increase)	0.00117 (increase)
Total brain volume	0.5475	0,0502	0,0524

Table S3. Analysis and comparison of detailed USV characteristics.

Different USV parameters by significance and impairment. "-" indicates no significant change as assessed by double-sided homoskedastic t-test. Arrows indicate a significant increase or a decrease of the respective parameter.

Nr2f1$^{+/-}$: Call Length (P4) P=0.0401, Slope (P4) P<0.0001 P8: Call Length (P8) P=0.0005, Frequency Delta (P8) P=0.0038, Sinuosity (P8) P=0.0033

Nr2f1$^{+/R139L}$: Latency to first (P4) P=0.0010, Time to last (P4) P=0.0010, Average call length (P4) P<0.0001, Highest frequency (P4) P=0.0166, Power (P4) P<0.0001, Tonality (P4) P<0.0001, Slope (P12) P=0.0137.

Nr2f1$^{+/E397*}$: Average call length (P4) P<0.0001, Frequency delta (P4) P=0.0036, Slope (P4) P=0.0213, Sinuosity (P4) P=0.0087, Power (P4) P=0.0006, Tonality (P4) P=0.0011, Slope (P12) P=0.0184.

Summary of vocalization parameters for different Nr2f1 genotypes across developmental stages (P4, P8, P12).

	P4			P8			P12		
Metric	Nr2f1$^{+/-}$	Nr2f1$^{+/R139L}$	Nr2f1$^{+/E397^*}$	Nr2f1$^{+/-}$	Nr2f1$^{+/R139L}$	Nr2f1$^{+/E397^*}$	Nr2f1$^{+/-}$	Nr2f1$^{+/R139L}$	Nr2f1$^{+/E397^*}$
Latency to first call	-	↑	-	-	-	-	-	-	-
Time of last call	-	↑	-	-	-	-	-	-	-
Average call duration	↓	↓	↓	↓	-	-	-	-	-
Principal Frequency (kHz)	-	-	-	-	-	-	-	-	-
Low Freq (kHz)	-	-	-	-	-	-	-	-	-
High Freq (kHz)	-	↓	-	-	-	-	-	-	-
Delta Freq (kHz)	-	-	↓	↓	-	-	-	-	-
Slope (kHz/s)	↓	-	↓	-	-	-	-	↓	↓
Sinuosity	-	-	↓	↓	-	-	-	-	-
Power (dB/Hz)	-	↓	↓	-	-	-	-	-	-
Tonality	-	↓	↓	-	-	-	-	-	-

Table S4. Overview of the number of significant results for each behavioral experiment for thethree mouse models.

USV (Ultrasonic Vocalization), EPM (Elevated Plus Maze), OF (Open Field), DLB (Dark Light Box), HB (Hole Board), SIT (Social Interaction Test), LB (Laboras), APA (Active Place Avoidance) and CW (CatWalk XT). Detailed USV characteristics (Table S3), brain morphology analysis (Table S2) and sex specific analysis are not included.

Experiment	***Nr2f1***$^{+/-}$	***Nr2f1***$^{+/R139L}$	***Nr2f1***$^{+/E397^*}$
Weight (all timepoints)	0	5	0
USV P4	2	2	2
USV P8	1	0	0
USV P12	0	1	1
EPM	0	3	1
OF	0	2	0
DLB	0	0	0
HB	0	0	0
SIT	0	4	1
LB	0	1	2
APA	0	4	2
CW	0	2	8
**Total Significant Results**	**3**	**24**	**17**

Disease Models & Mechanisms • Supplementary information

Table S5. Comparison of the mouse models by effect size (Cohen's d).

Several parameters were significant in more than one line. Adding to the quantitative comparison we evaluated the effect size using Cohen's *d*. For each parameter and mouse model (if the result was significant as tested by Student's t-test), we calculated *d*. On average, *d* was 0.6810, 1.1035, and 1.0054 for *Nr2f1*$^{+/-}$, *Nr2f1*$^{+/R139L}$, and *Nr2f1*$^{+/E397*}$ mice, respectively.

Parameter	*Nr2f1*$^{+/-}$	*Nr2f1*$^{+/R139L}$	*Nr2f1*$^{+/E397^*}$
Weight P4 Males	1.5715	3.0305	1.6667
USV P4 Number of Calls	0.5808	1.7893	1.6818
USV P4 Complex vs Simple Calls	0.3578	0.8456	0.6307
USV P12 Complex vs Simple Calls	ns	0.4685	0.4664
EPM Visits Open Arms	ns	0.7818	0.6155
APA Shocks During Training	ns	0.8890	1.4136
CW BOS Width Between Front Paws	0.4238	0.5844	0.7054
CW RF - LF Coupling	0.4712	0.4386	0.5632
**Average effect size (d)**	**0.6810**	**1.1035**	**1.0054**

Disease Models & Mechanisms • Supplementary information

Table S6. List of all p-values by Student's t-test for the behavioral tests.

USV (Ultrasonic Vocalization), EPM (Elevated Plus Maze), OF (Open Field), DLB (Dark Light Box), HB (Hole Board), SIT (Social Interaction Test), LB (Laboras), APA (Active Place Avoidance) and CW (CatWalk XT). The table shows the p values for the different tests and parameters as calculated by the Student's T-test. Outliers (> 3 standard deviations from the mean) were not included in the T-test. For details see Methods; Behavioral Data Analysis and Statistics.

Table 1 – fortgesetzt

Parameter	*Nr2f1*$^{+/-}$	*Nr2f1*$^{+/R139L}$	*Nr2f1*$^{+/E397^*}$
**Weight P4**	0.8104	0.0111	0.3407
**Weight P8**	0.9495	0.0224	0.4089
**Weight P12**	0.6128	0.0365	0.4776
**Weight P20**	0.5658	0.0176	0.3443
**Weight P30**	0.9922	0.6604	0.4286
**Weight P100**	0.2422	0.0336	0.0638
**USV P4**			
Number of Calls	0.0066	<0.0001	<0.0001
Complex vs Simple Calls	0.0166	<0.0001	0.0040
**USV P8**			
Number of Calls	0.4739	0.6568	0.8930
Complex vs Simple Calls	0.0395	0.2269	0.3239
**USV P12**			
Number of Calls	0.1316	0.9095	0.8475
Complex vs Simple Calls	0.3882	0.0124	0.0322
**EPM**			
Time in Closed	0.6753	0.2455	0.3812
Time in Middle	0.2075	0.3099	0.6628
Time in Open Arms	0.7473	0.0370	0.3812
Total Distance	0.5786	0.0060	0.3184
Visits Open Arms	0.5516	0.0223	0.0398
**OF**			
Time in Middle	0.9294	0.0012	0.5850
Total Distance	0.0678	0.0074	0.1629
**DLB**			
Delay to First Entry	0.5691	0.5371	0.1710
Total Distance	0.6516	0.1328	0.4154
Visits	0.3424	0.9984	0.1699
Time in Light Area	0.6647	0.5087	0.4077
Distance in Light Area	0.8302	0.6736	0.4971
**HB**			
Nose Pokes	0.9917	0.1316	0.0764
**SIT Trial 2**			
Time with Intruder	0.3257	0.7458	0.3356
Time with Littermate	0.6366	0.9562	0.1261
Time in Close Contact with Intruder	0.4382	0.2599	0.7604
Time in Close Contact with Littermate	0.2794	0.7362	0.4893

Parameter	***Nr2f1***$^{+/-}$	***Nr2f1***$^{+/R139L}$	***Nr2f1***$^{+/E397^*}$
**SIT Trial 3**			
Time with Intruder	0.3843	0.0119	0.7215
Time with Littermate	0.2516	0.0196	0.3193
Time in Close Contact with Intruder	0.3898	0.0186	0.5331
Time in Close Contact with Littermate	0.7939	0.2411	0.0230
Sociability Index	0.3148	0.0178	0.3303
**LB**			
Climbing Frequency	0.5428	0.0468	0.5406
Climbing Duration	0.5529	0.1079	0.0373
Locomotion Duration	0.1138	0.8262	0.0358
**APA**			
Recall Trial; Time in Zone	0.9306	0.0315	0.6369
Recall Trial; Number of Shocks	0.7526	0.0371	0.3954
Training Trial; Time in Zone	0.3166	0.0470	0.0166
Training Trial; Number of Shocks	0.2386	0.0218	0.0004
Latency to First Shock During Recall	0.8263	0.1668	0.6369
**CW**			
Width Between Front Paws	0.3052	0.0214	0.0004
Couplings_RF→LH_CStat_Mean	0.1021	0.0596	0.0375
Couplings_LF→RH_CStat_Mean	0.2868	0.3978	0.0234
Couplings_RH→LF_CStat_Mean	0.2859	0.6792	0.0026
Couplings_LF→RF_CStat_Mean	0.4148	0.1293	0.0161
Couplings_RF→LF_CStat_Mean	0.9217	0.0392	0.0430
Couplings_LF→LH_CStat_Mean	0.4784	0.1404	0.0001
Couplings_LH→LF_CStat_Mean	0.3037	0.0962	0.0001
Speed	0.6781	0.8498	0.9068
Width between hind paws	0.1426	0.5551	0.9586
Number of step sequence patterns	0.7395	0.1980	0.1840
Support gridle paws	0.4326	0.4378	0.1069
Support diagonal paws	0.6775	0.8430	0.5614
Regularity Index	0.2748	0.1034	0.6433
P_{LDA}	0.3316	0.3889	0.7507

Table S7. Overview of cohort sizes and sex distribution across behavioral tests. This table summarizes the number of male and female mice, as well as the number of litters, tested at each stage of the behavioral pipeline for each mouse line. The tests include: USV (Ultrasonic Vocalizations), EPM (Elevated Plus Maze), OF (Open Field), DLB (Dark-Light Box), HB (Hole Board), SIT (Social Interaction Test), LB (Laboras), APA (Active Place Avoidance), and CW (CatWalk XT). All animals had identical experimental histories at each timepoint of testing. No new animals were added to any cohort, and wild-type and mutant mice were always tested in parallel. Testing was performed in two cohorts, consisting of 66 and 142 animals, respectively. For details see Methods;Animals.

Test	Line	Genotype	Males	Females	Total	Litters
USV P4	KO	WT	25	23	48	13
		$Nr2f1^{+/-}$	24	20	44	
	R139L	WT	22	26	48	12
		$Nr2f1^{+/R139L}$	14	11	25	
	E397*	WT	18	8	26	9
		$Nr2f1^{+/E397*}$	10	7	17	
USV P8	KO	WT	24	22	46	13
		$Nr2f1^{+/-}$	19	22	41	
	R139L	WT	21	24	45	10
		$Nr2f1^{+/R139L}$	13	10	23	
	E397*	WT	18	8	26	9
		$Nr2f1^{+/E397*}$	10	7	17	
USV P12	KO	WT	19	20	39	13
		$Nr2f1^{+/-}$	21	19	40	
	R139L	WT	20	22	42	10
		$Nr2f1^{+/R139L}$	13	10	23	
	E397*	WT	18	8	26	9
		$Nr2f1^{+/E397*}$	11	5	16	
EPM	KO	WT	7	6	13	5
		$Nr2f1^{+/-}$	7	7	14	
	R139L	WT	18	20	38	8
		$Nr2f1^{+/R139L}$	8	4	12	
	E397*	WT	19	9	28	8
		$Nr2f1^{+/E397*}$	11	10	21	
OF	KO	WT	7	7	14	5
		$Nr2f1^{+/-}$	8	6	14	
	R139L	WT	13	12	25	8
		$Nr2f1^{+/R139L}$	8	6	14	
	E397*	WT	20	8	28	8
		$Nr2f1^{+/E397*}$	10	10	20	
DLB	KO	WT	7	7	14	5
		$Nr2f1^{+/-}$	8	6	14	
	R139L	WT	13	12	25	8
		$Nr2f1^{+/R139L}$	8	6	14	
	E397*	WT	18	8	26	8
		$Nr2f1^{+/E397*}$	10	6	16	

**Test**	**Line**	**Genotype**	**Males**	**Females**	**Total**	**Litters**
HB	KO	WT	7	7	14	5
		$Nr2f1^{+/-}$	8	7	15	
	R139L	WT	13	12	25	8
		$Nr2f1^{+/R139L}$	8	6	14	
	E397*	WT	15	8	26	8
		$Nr2f1^{+/E397*}$	10	5	16	
SIT	KO	WT	7	7	14	5
		$Nr2f1^{+/-}$	8	7	14	
	R139L	WT	9	9	18	5
		$Nr2f1^{+/R139L}$	6	6	12	
	E397*	WT	10	7	17	5
		$Nr2f1^{+/E397*}$	11	5	16	
CW	KO	WT	7	7	13	5
		$Nr2f1^{+/-}$	8	8	16	
	R139L	WT	9	9	18	5
		$Nr2f1^{+/R139L}$	8	6	14	
	E397*	WT	10	9	19	5
		$Nr2f1^{+/E397*}$	10	7	17	
APA	KO	WT	7	7	13	5
		$Nr2f1^{+/-}$	8	8	16	
	R139L	WT	9	8	17	5
		$Nr2f1^{+/R139L}$	8	6	14	
	E397*	WT	10	6	16	5
		$Nr2f1^{+/E397*}$	10	6	16	

Disease Models & Mechanisms • Supplementary information
